# Dynamics of diazotroph particle colonization in the Arctic Ocean

**DOI:** 10.1093/ismejo/wraf098

**Published:** 2025-05-20

**Authors:** Arthur Coët, Cécile Carpaneto Bastos, Mathias Lechelon, Ruth Hawley, Oliver Flanagan, Maeve C Lohan, Pierre Ronceray, Joanne E Hopkins, Claire Mahaffey, Mar Benavides

**Affiliations:** Aix Marseille Univ, Université de Toulon, CNRS, IRD, MIO UM 110, Marseille, France; Aix Marseille Univ, CNRS, CINAM, Turing Centre for Living Systems, Marseille, France; Aix Marseille Univ, Université de Toulon, CNRS, IRD, MIO UM 110, Marseille, France; Aix Marseille Univ, CNRS, CINAM, Turing Centre for Living Systems, Marseille, France; Aix Marseille Univ, CNRS, INSERM, MEP Centuri, Turing Centre for Living Systems, Marseille, France; Department of Ocean and Earth Sciences, University of Southampton, National Oceanography Centre, Southampton, SO14 3ZH, United Kingdom; Department of Ocean and Earth Sciences, University of Southampton, National Oceanography Centre, Southampton, SO14 3ZH, United Kingdom; Department of Ocean and Earth Sciences, University of Southampton, National Oceanography Centre, Southampton, SO14 3ZH, United Kingdom; Aix Marseille Univ, CNRS, CINAM, Turing Centre for Living Systems, Marseille, France; National Oceanography Centre, Liverpool L3 5DA, United Kingdom; Department of Earth, Ocean, and Ecological Sciences, University of Liverpool, Liverpool, United Kingdom; Aix Marseille Univ, Université de Toulon, CNRS, IRD, MIO UM 110, Marseille, France; National Oceanography Centre, European Way, Southampton SO14 3ZH, United Kingdom

**Keywords:** nitrogen fixation, organic matter, chemotaxis, Barents Sea

## Abstract

Global warming is causing sea ice retreat and intensifying algal blooms in the Arctic Ocean, in turn increasing nitrogen limitation in surface waters. Dinitrogen fixation by diazotrophic microorganisms, usually favored in low reactive nitrogen systems, may become an increasingly important source of nitrogen in the Arctic. Previous studies have shown that non-cyanobacterial diazotrophs are dominant in the Arctic Ocean. Lacking a photosynthetic apparatus, non-cyanobacterial diazotrophs may utilize organic particles as carbon- and energy-rich niches. However, cyanobacterial diazotrophs may also form particles by aggregation. To further understand diazotroph-particle associations, here we study the chemotactic behavior and colonization dynamics of diazotrophs on model organic particles using a modified chemotaxis assay. Artificial organic particles (agarose, alginate) were incubated with surface seawater communities from four contrasted stations in the Barents Sea, and their DNA was sequenced targeting *nifH* and 16S rRNA genes after 2, 36, and 72 h of incubation. Our results show that diazotroph groups have selective colonization behaviors, with *Gammaproteobacteria* members preferentially colonizing alginate particles derived from brown algae, a form of organic matter becoming more common in the Arctic as it warms up. We also observe niche partitioning among microbial groups, with diazotrophs colonizing nitrogen-poor, carbon-rich particles earlier than non-diazotrophic prokaryotes. As Arctic warming proceeds, increased algal blooms may expand the niches for particle-associated diazotrophs, whose dinitrogen fixation supports phytoplankton growth and primary productivity.

## Introduction

Global warming is driving significant transformations in the Arctic Ocean [1, 2] triggering profound shifts in its biogeochemistry, nutrient cycles, and primary production through coastal morphology changes and diminishing ice cover [3, 4]. The increasingly early seasonal ice retreat [5], potentially leading to ice-free summers by 2050 [6], catalyzes a cascade of biogeochemical alterations [7]. This dramatic transformation unleashes nutrients previously sequestered beneath the ice [8] allowing deeper light penetration, ultimately stimulating algal and phytoplankton proliferation [9]. These blooms deplete bioavailable nitrogen, leading to surface nitrogen limitation [10, 11]. In other nitrogen-limited systems such as subtropical gyres, dinitrogen (N_2_) fixation represents a major source of bioavailable nitrogen [12]. As the Arctic becomes increasingly nitrogen-limited, N_2_ fixation is expected to become a key source of bioavailable nitrogen in this region as well [13]. These changes in nitrogen availability, coupled with increasing organic carbon input from algal bloom and ice melt, are reshaping microbial community dynamics and biogeochemical cycles in the Arctic Ocean [14].

N_2_ fixation is facilitated by nitrogenase, an oxygen-sensitive enzyme found in a group of microorganisms called “diazotrophs” [12]. Cyanobacteria have traditionally been regarded as the primary diazotrophs in marine environments [15]. However, growing evidence shows that non-cyanobacterial diazotrophs (NCDs) dominate not only in low latitudes but also in coastal, deep, and polar oceans [13, 16, 17]. Despite their widespread distribution, there are uncertainties about the N_2_ fixation potential of NCDs [18, 19]. In contrast to cyanobacteria, NCDs are unable to do photosynthesis and are thought to rely on particulate organic matter to obtain carbon and energy [20–22]. However, diazotrophic cyanobacteria may also rely on dissolved organic matter under nitrogen limitation or environmental stress [23, 24], suggesting that cyanobacterial diazotrophs might increase its organic matter dependence in the changing Arctic [25].

Marine particles, commonly known as “marine snow”, are aggregates of organic and inorganic material such as phytoplankton, algae, zooplankton excretion, cellular debris, and sediment [26]. These aggregates play a pivotal role in the marine carbon cycle by transporting captured carbon dioxide from the atmosphere to the deep ocean, reducing atmospheric CO_2_ levels (the “biological carbon pump”; [27, 28]). Additionally, marine particles serve as hotspots of microbial activity where carbon and nitrogen cycles are tightly coupled through diverse microbial metabolisms [22, 29]. Particles are considered favorable loci for NCDs, provided by their labile carbon content and low oxygen microniches [22, 30, 31]. Although *nifH* gene sequences (encoding for a subunit of the nitrogenase enzyme) are frequently detected in particle-associated fractions (e.g., >3 μm; [32–34]), the specific mechanisms governing these associations remain poorly understood [22]. With increasing nitrogen limitation and changing particle flux in the Arctic [35], understanding NCDs interactions with particles is crucial for predicting reactive nitrogen supply in future conditions.

Particles in the Arctic Ocean originate from diverse sources, including phytoplankton, algae, and glacial runoff [14, 36, 37]. Reduced ice cover increases the input of fresh organic matter, thereby altering the composition and behavior of the resulting particulate matter and organic aggregates [38]. Moreover, recent studies have shown that melting Arctic sea ice significantly influences carbon export through the ballasting effect of cryogenic gypsum and terrigenous materials [39]. These particle dynamics are distinctive of the Arctic environment, making it an ideal location for investigating their complex interactions with diazotrophs.

Planktonic microorganisms use chemotaxis to colonize particles, moving along chemical gradients [40]. This process helps microorganisms locate optimal environments, especially for carbon sources, and shapes microbial community assembly on marine particles, a behavior observed in various bacterial groups including marine diazotrophs [41–43]. However, the interplay between carbon availability and nitrogen metabolism in driving particle colonization is poorly understood. Here we hypothesized that the composition of particles (e.g., derived from brown vs. red algae) may favor different diazotroph groups due to their carbon and energy requirements to fuel N_2_ fixation. We further propose that diazotroph colonization strategies might differ from non-diazotrophs, as NCDs can meet nitrogen needs via N_2_ fixation, potentially giving them an advantage in colonizing nitrogen-poor, carbon-rich particles. Following the major phytoplankton bloom detected by TOPAZ5-ECOSMO satellite data in June 2023 (Fig. S1), our study explores how diazotrophs interact with marine particles amid Arctic ecosystem changes. Using an adapted chemotaxis assay [44] deployed across four contrasting sampling stations and *nifH*/16S rRNA gene sequencing, we assessed whether diazotroph groups show distinct colonization preferences on different polysaccharide particles and how environmental conditions affect these interactions. Our findings shed light on the interactions between diazotrophs and particles, which may be increasingly relevant as ice retreats, expanding diazotroph niches and enriching the particle loading of the Arctic pelagic system.

## Materials and methods

### Chemotaxis assay

**Figure 1:**
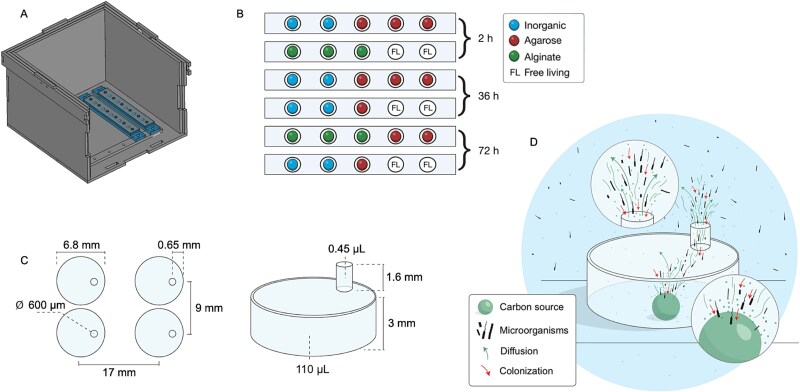
Overview of the modified ISCA model used on the RSS discovery. Schematic of the modified ISCA design mounted on the base of a PMMA chamber, facilitating controlled incubations. STEP files used for this 3D design are available in the supplementary materials and parametric FCStd files for device modification are available on the github repository (A). Diagram of the ISCA wells, highlighting the placement of various artificials particles (alginate and agarose in triplicate) and controls (inorganic and free-living in duplicate), along with the assigned incubation durations (B). Detailed close-up of the wells, specifying their dimensions and unique features (C). Simplified illustration of the dynamics of diffusion, chemotaxis, and colonization within the wells during the incubation process (D).

**Figure 2:**
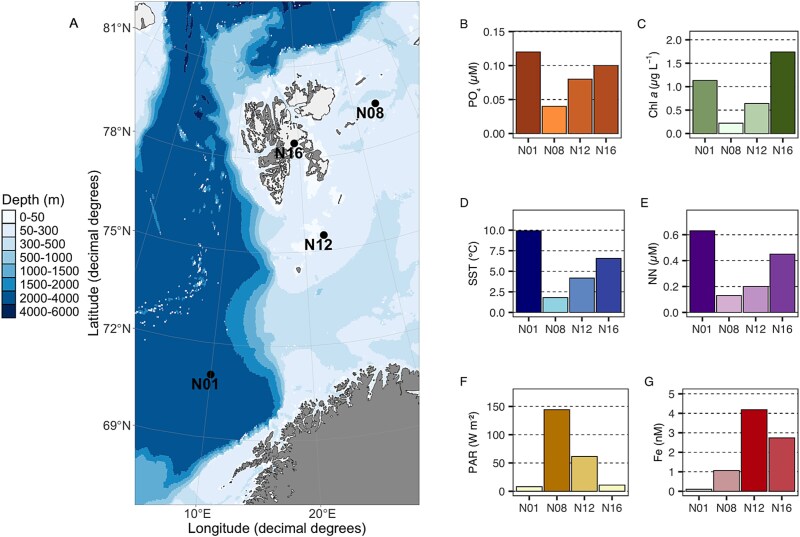
Bathymetric map of the sampling stations (N01, N08, N12, N16) in the Barents Sea (A), accompanied by bar charts illustrating the concentrations of various environmental parameters measured at the stations. Depths are represented by a color gradient ranging from light blue (0–50 m) to dark blue (4000–6000 m). The bar charts display variations in: (B) sea surface temperature (SST), (C) nitrates and nitrites (NN), (D) phosphate (PO_4_^3−^), (E) chlorophyll *a* (Chl *a*), (F) photosynthetically active radiation (PAR), and (G) iron (Fe), across the stations. Each station shows distinct gradients, indicating heterogeneity in environmental conditions across the sampling sites. These measurements were conducted without replication.

Four distinct sampling stations were selected for diazotroph chemotaxis experiments using a custom made modified version of the in situ chemotaxis assay (ISCA; Fig. 1). Based on water mass definitions [45], surface waters were predominantly characterized as warm Polar Water, except for station N01 located in Atlantic Water in the deep Norwegian Basin (70.59°N, 10.59°E). Station N12 was situated on the shallow, well-mixed Spitzbergen Bank (75.30°N, 22.29°E), influenced by a mixture of Polar and Atlantic waters. Station N08 was characterized by seasonal ice-melt water east of Svalbard (79.20°N, 33.58°E), and station N16 was influenced by glacial melt at the northern end of Storfjorden (78.50°N, 19.26°E) (Fig. 2, Fig. S2). Surface waters showed distinct physical properties, with temperatures ranging from 2.5 to 11°C and salinities from 32.5 to 35 g kg^−1^ (Fig. S2).

The ISCA (Fig. 1A, S6) contains multiple bars (Fig. 1B) with five 3D-printed wells each. Wells hold agarose, alginate, glass beads, or remain non-inoculated. Each well (Fig. 1C, S6) has a lid with a 600 μm hole allowing chemical diffusion without cross-contamination. The design (Fig. 1D) enables chemical cues from artificial particles to attract and facilitate microbial colonization. Additional technical details appear in Supplementary Methods.

Artificial particles included inorganic controls (glass) and organic particles (agarose and alginate) (Supplementary Methods). Alginate and agarose were selected for their prevalence in marine ecosystems (brown and red algae, respectively) [46, 47], and their ecological relevance in Arctic coastal waters as bacterial substrates [47, 48]. Before incubations, artificial particles were rinsed with filtered seawater and measured with a Dino-Lite digital microscope (200X magnification, 640 x 480 pixels; Hsinchu, Taiwan). Six lanes of five wells containing artificial organic and inorganic particles with empty controls for free-living organisms (Fig. 1) were fixed to the ISCA incubator base. A funnel was used to add 4 L of 10 μm-filtered seawater, preventing air bubbles. The incubations were conducted in triplicate for each artificial particle with inorganic and free-living controls added in duplicate (Fig. 1). The incubation of the ISCA was carried out in an incubator set to the temperature and light conditions present in the surface water (5 m) at each station (Fig. 2). Particles and free-living samples were collected at 2, 36, and 72 h and preserved in 50% ethanol-filtered seawater solution [49]. The samples were then stored at −80°C until further analysis. DNA was extracted from individual particles and particle-free wells at each sampling time point and sequenced for *nifH* and 16S rRNA genes (Supplementary methods).

### Statistics

Statistical analyses were performed using R version 4.4.1. ASV counts for free-living communities (110 μL) were normalized to match particle sample volumes (0.5 μL), calculated based on the average diameter (1 mm) of the artificial particles. Relative abundance of each taxonomic class was calculated as percentage of total ASVs per sample. To handle the high variability in abundance between taxa and focus on relative changes between treatments, data were log-transformed. Following transformation, data normality was assessed using Shapiro–Wilk (SW) test. Due to persistent non-normal distribution, Mann–Whitney U-tests (MW) were conducted for each sampling station and for each taxonomic class of interest. The model compared relative abundances between carbon sources (agarose vs. alginate). *P* values were adjusted using the Benjamini-Hochberg correction.

For colonization dynamics, temporal patterns were further standardized by normalizing the maximum relative abundance observed across the three time points (2, 36, and 72 h) to 100% for each microbial class, station, and carbon source. The relative abundances at all other incubation times were then expressed as a percentage of this maximum value. Bootstrap confidence intervals (n = 1000, α = 0.05) assessed abundance measurement uncertainty. Kruskal-Wallis tests followed by Bonferroni-corrected pairwise Wilcoxon rank sum tests evaluated temporal colonization pattern variations. This approach enabled the quantification of temporal changes in colonization patterns for each microbial class, station, and artificial particle type.

Fold changes were calculated using the initial time point as the reference for both *nifH* and 16S rRNA genes datasets. To compare colonization patterns between particle types, Fligner–Killeen’s test was used to assess variance homogeneity, followed by Kruskal–Wallis, and pairwise Mann–Whitney U-tests to determine significant differences in fold changes at each time point. This approach was applied to both diazotrophic and total bacterial communities to assess their respective colonization dynamics.

## Results

### Diazotroph and bulk prokaryotic community composition

We assigned 16S rRNA and *nifH* gene amplicon sequences to the class level to compare the diversity of bulk and diazotrophic microbial communities. From the 120 samples collected, only 94 and 52 yielded sufficient amplification of the 16S rRNA and *nifH* gene, respectively (Table S3, S4, S7, S8). The low concentration of extracted DNA (< 2 ng μL^−1^) may have contributed to the reduced number of successful amplifications. A total of 1 462 209 reads were generated from the 16S rRNA gene sequencing, resulting in 1119 ASVs. The taxonomic breakdown included 19 phyla, 34 classes, 80 orders, 134 families, and 179 genera (Table S5). In contrast, *nifH* sequencing yielded 273 993 reads and 999 ASVs, encompassing five phyla, eight classes, 15 orders, 14 families, and 14 genera (Table S6). The free-living fraction served as a reference for normalization and represented the available seed bank community (Fig. S7), allowing identification of active colonizers beyond their initial presence in surrounding water. *nifH* sequences were predominantly assigned to families within *Alphaproteobacteria*, “*Betaproteobacetria*”, and *Gammaproteobacteria* classes, as well as *Cyanophyceae*, including *Alcaligenaceae,* and orders like *Hyphomicrobiales.* In contrast, the 16S rRNA gene dataset revealed a more diverse bacterial community (Tables S5, S6). We used 16S rRNA gene sequence abundance to represent non-diazotrophs, as diazotrophs typically comprise less than 1% of the bacterial community in the small size fraction (<10 μm) [50]. Class-level taxonomy was used for all analyses to directly compare 16S rRNA gene and *nifH* datasets, as genus-level analysis of 16S rRNA gene showed few substrate preferences differences and *nifH* lacked sufficient depth for genus-level resolution. Family level analyses were also performed but revealed higher variability between replicates and taxonomic discordance between *nifH* and 16S rRNA genes datasets, potentially masking ecological signals (Fig. S8). Although the term “*Betaproteobacteria*” is no longer formally recognized in current bacterial taxonomy, we retain its use here for consistency with the *nifH* database classification.

### Environmental settings

Environmental conditions varied among stations (N01, N08, N12, N16), reflecting Barents Sea heterogeneity (Fig. 2). Sea surface temperature showed a latitudinal gradient from 9.92°C (N01) to 1.79°C (northernmost N08) (Fig. 2D; Fig. S2). Phosphate and nitrate + nitrite (PO4 and NN; Fig. 2B and 2E, respectively) had a comparable pattern across stations, aligning with the distribution of SST and Chl *a* (Fig. 2B,C). Atlantic-influenced stations (N01, N16) had highest nutrients (NN: 0.63, 0.45 μM; PO4: 0.12, 0.04 μM), elevated temperatures (9.92°C, 6.56°C), and Chl *a* concentrations (1.13, 1.74 μg L^−1^), indicating productive waters. In contrast, station N08 had the lowest nutrient (NN: 0.13 μM, PO4: 0.02 μM) and Chl *a* concentrations (0.22 μg L^−1^), indicative of more relatively oligotrophic conditions. The higher nutrient concentrations at station N16 situated in proximity to the Storfjorden glacier are likely attributable to glacial meltwater inputs (Fig. S2), supporting high Chl *a* concentrations with low photosynthetic active radiation (PAR: 62.71 W m^−2^; Fig. 1F). This suggests that phytoplankton biomass reduced light penetration deeper into the water column at this station. In contrast with the observed trends for macronutrients, the highest measurable concentrations of dissolved iron (dFe; Fig. 1G) were observed at stations N12 and N16 (4.18 and 2.74 nM, respectively), with potentially higher concentrations at nearby stations. In contrast, dFe concentrations at station N01 (0.10 nM), influenced by Atlantic waters, were approximately 40 times lower than at station N12. Distinct nutrient and iron gradients across stations provided an ideal setting to examine how environmental factors shape microbial communities and particle colonization dynamics.

### Dynamics of diazotroph particle colonization

Different diazotroph groups exhibited distinct colonization patterns across particle types. Although inorganic particles showed minimal variance changes typical of passive colonization (Fig. S3d), both organic substrates demonstrated evidence of active colonization processes, although through different patterns. Agarose particles showed consistent colonization patterns with progressively decreasing variance over time, suggesting a convergent community assembly process (Supplementary Results). In contrast, alginate particles exhibited a more dramatic temporal heterogeneity (mean fold changes: 12.1 → 3.73 → 12.3) with heterogeneous variances (0.247 → 2.50 → 0.610; Fligner-Killeen test: χ^2^ = 10.226, *P* < 0.01), indicating highly dynamic colonization processes (Supplementary Results).

**Figure 3:**
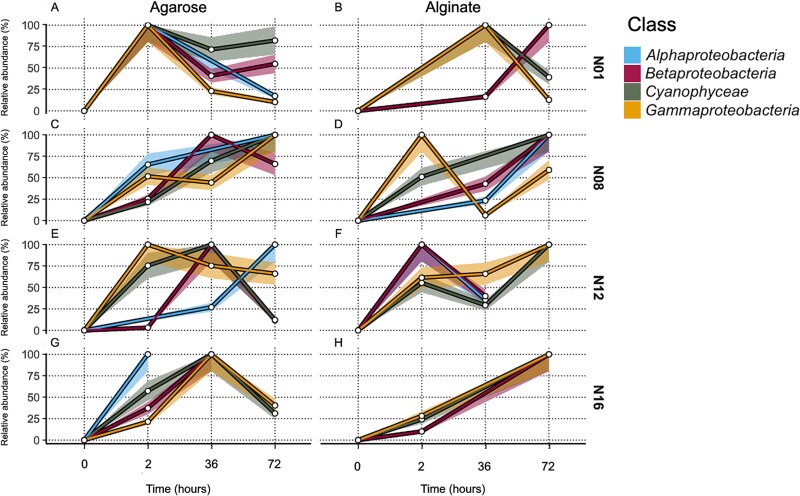
Normalized relative abundance of diazotrophs groups over time in agarose (A,C,E,G) and alginate (B,D,F,H) artificial particles at four Barents Sea stations (N01, N08, N12, N16). The diazotroph groups include representatives of the *Gammaproteobacteria*, *Cyanophyceae*, “*Betaproteobacteria”*, and *Alphaproteobacteria*. The time points included in the study were 0, 2, 36, and 72 h. Bootstrap confidence intervals (95%) are displayed as semi-transparent shaded areas around each curve. The number of samples included in each data point are provided in Table S7.

*Gammaproteobacteria* members colonized agarose particles rapidly at stations N01 and N12, reaching 100% relative abundance within 2 h (Fig. 3A, E) (KW, *P* < 0.05). This was followed by a systematic succession of other groups, suggesting an organized colonization process driven by substrate-specific interactions. “*Betaproteobacteria”* representatives showed sequential colonization peaks at stations N08, N12, and N16, reaching 100% abundance at 36 h or later (Fig. 3C-E, G-H) with significant variations at N08 and N12 (KW, *P* < 0.05), while *Alphaproteobacteria* members increased steadily at stations N08 (KW, *P* < 0.05) and N12 (KW, *P* < 0.05) reaching 100% by 72 h (Fig. 3C, E). The colonization dynamics for *Alphaproteobacteria* taxa at stations N01 and N16 could not be assessed due to lack of *nifH* amplification at several time points (Table S3).

The colonization patterns on alginate particles were more variable, characterized by a swift initial colonization of *Gammaproteobacteria* at stations N01, N08, and N16 (KW, *P* < 0.05) (Fig. 3B, D, H), followed by substantial temporal fluctuations in community composition. Other diazotroph groups showed less predictable patterns of colonization, particularly at station N12 where *Alpha*- and “*Betaproteobacteria”* declined abruptly after 2 h (KW, *P* < 0.05) (Fig. 3F). The absence of *Alphaproteobacteria* on alginate particles at stations N01 and N16, coupled with their variable colonization patterns, indicates substrate-specific selection processes.

### Carbon source preferences

**Figure 4:**
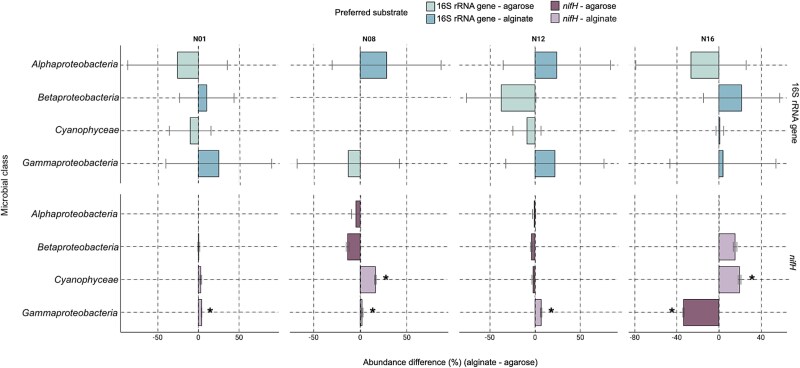
Barplot showing the relative abundance of microbial classes associated with alginate and agarose artificial particles at stations N01 (A, B), N08 (C, D), N12 (E, F), and N16 (G, H) for both the 16S and *nifH* genes. The bars represent the difference in relative abundance between the two carbon sources, with positive values indicating a preference for alginate and negative values indicating a preference for agarose. The microbial classes shown include *Alphaproteobacteria*, “*betaproteobacteria”*, *Gammaproteobacteria*, and *Cyanophyceae*. Error bars indicate the standard deviation for each substrate. Asterisks (*) indicate statistically significant differences between substrates (*P* < 0.05). The number of samples included in each data point are provided in Table S7.

To investigate carbon source preferences among taxa, we analyzed the relative abundance of taxa at the class level on alginate and agarose artificial particles, examining diazotrophic (*nifH*) and total prokaryotic communities (16S RNA gene) separately (Tables S1, S2). Within diazotrophic communities *Gammaproteobacteria* members demonstrated a clear preference for alginate over agarose at most stations, except for station N16. At station N01, the relative abundance of diazotrophic *Gammaproteobacteria* taxa on alginate particles reached 48%, significantly higher than on agarose (MW, *P* < 0.05; Fig. 4). Higher preferences for alginate compared to agarose were observed too at station N08 (34% vs. 32%; MW, *P* < 0.05) and station N12 (26% vs. 19%; MW, *P* < 0.05). However, at station N16 members of diazotrophic *Gammaproteobacteria* exhibited a reversed preference showing significantly higher abundance on agarose (52%) as compared to alginate (18.5%; MW, *P* < 0.05; Fig. 4). Diazotrophic *Cyanophyceae* representatives exhibited varying substrate associations across stations. At stations N08 and N16, they showed a significant preference for alginate over agarose (42.5% vs. 26.2%, MW, *P* < 0.001; 56.8% vs. 37.1%, MW, *P* < 0.001 respectively; Fig. 4, Table S1), while showing no significant substrate preference at stations N01 and N12. Diazotrophic *Alphaproteobacteria* taxa were either undetected on alginate particles at stations N01 and N16 or showed no significant carbon source preferences at stations N08 and N12 (Table S1, Fig. 3D,F). Diazotrophic “*Betaproteobacteria”* members showed no significant substrate preference across stations (MW, *P* > 0.05), with abundances ranging from 8.31–23.9% on agarose and 8.90–24.8% on alginate.

Analysis of the bulk prokaryotic community revealed high variability across substrates and stations, with no taxonomic groups showing no significant preference for either carbon source (Table S2; Fig. 3). All major taxonomic groups displayed high variability in their relative abundances both between stations and between replicates, with differences ranging from 1% to over 70% for some groups. For example, *Alphaproteobacteria* abundances varied from 14.4% to 68.9% on alginate and from 27.3% to 43.9% on agarose across stations, whereas *Gammaproteobacteria* showed similarly wide ranges (18.8–72.4% on alginate, 31.8–43.9% on agarose). Although substantial variations in relative abundances were observed, these differences did not reach statistical significance (all *P* > 0.05). Unlike the diazotrophic community, where *Gammaproteobacteria* and *Cyanophyceae* showed significant substrate preferences across multiple stations, their non-diazotrophic counterparts displayed no such selectivity.

### Bulk prokaryotic community particle colonization dynamics

**Figure 5:**
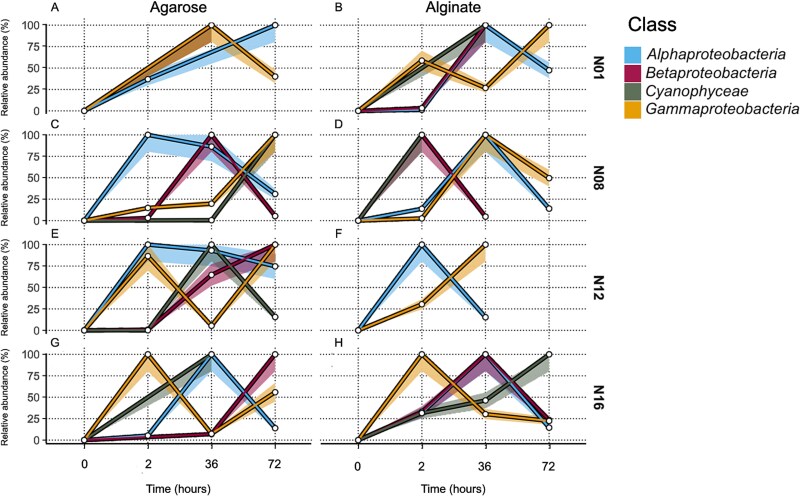
Normalized relative abundance of bulk microbial groups over time in agarose (A,C,E,G) and alginate (B,D,F,H) artificial particles at four Barents Sea stations (N01, N08, N12, N16). The bulk microbial groups include representatives of the *Gammaproteobacteria*, *Cyanophyceae*, “*betaproteobacteria”*, and *Alphaproteobacteria*. The time points included in the study were 0, 2, 36, and 72 h. Bootstrap confidence intervals (95%) are displayed as semi-transparent shaded areas around each curve. The number of samples included in each data point are provided in Table S8.

The bulk prokaryotic community exhibited colonization dynamics distinct from those of diazotrophs. Although maintaining similar substrate preferences, with more complex interactions on organic particles compared to passive accumulation on inorganic ones (Fig. S4, Supplementary Results), the temporal patterns and taxonomic succession differed. Indeed, both agarose and alginate particles exhibited active colonization signatures, suggesting substrate-specific selection processes (Supplementary Results). Contrary to the members of diazotrophic *Gammaproteobacteria*, the bulk prokaryotic *Gammaproteobacteria* ASVs were not the fastest particle colonizers, the faster group being *Alphaproteobacteria* taxa (Fig. 5A, C, G, H). These results indicate that although diazotrophic *Gammaproteobacteria* members initially dominate in both particle types, they do not maintain this predominance over time according to the 16S rRNA gene amplicon data. It appears that differences in colonization dynamics are slowing the overall colonization process, delaying the establishment of certain classes as other classes begin to emerge. For instance, the two-fold decrease in *Gammaproteobacteria* members on agarose at Station N01 between 36 and 72 h (Fig. 5A) coincided with a substantial increase in the relative abundance of *Alphaproteobacteria* representatives over the same period. This pattern was reversed on alginate particles at the same station (Fig. 5B). Certain classes appeared to coexist on the same carbon source with similar colonization dynamics. The colonization patterns of *Gammaproteobacteria* and *Cyanophyceae* taxa at station N08 provide an illustrative example of this (Fig. 5C). In contrast, on alginate particles at the same station, these two classes seemed to engage in competitive interactions. The rapid decline of *Cyanophyceae* ASVs between 2 and 36 h (from 100% to 0%) coincided with a sharp increase in Gammaproteobacteria ASVs (from 2 to 100%) during the same period (Fig. 5D). In line with these observations both *Gamma-* and *Alphaproteobacteria* members demonstrated similar colonization dynamics on alginate particles, albeit at different stations. At station N08 these two classes showed analogous patterns of colonization (Fig. 5D), suggesting a potential overlap in their ecological roles despite being observed at separate locations. At station N12, *Alphaproteobacteria* and *Gammaproteobacteria* taxa competed on alginate: *Alphaproteobacteria* ASVs declined from 100% to 14.9% while *Gammaproteobacteria* ASVs reached 100% between 2–36 h (Fig. 5F).

## Discussion

### Niche partitioning drives diazotroph succession on particles

Particle-associated diazotrophic communities showed clear succession patterns, following classical ecological succession theory where early colonizers modify the environment for subsequent colonizers [51]. Transitions from rapid colonizers to secondary resource specialists align with the tolerance model, where later colonizers exploit altered conditions independently [52]. Across different stations and carbon sources we observed an initial phase dominated by rapid colonizers followed by a gradual shift towards taxa better adapted to exploit the byproducts of earlier colonizers, demonstrating both niche partitioning and facilitation mechanisms [53]. Sequential colonization indicates that the environmental biogeochemical conditions and species competition shape particle communities, affecting organic matter cycling and N_2_ fixation by particle-associated diazotrophs.

*Gammaproteobacteria* representatives colonized agarose particles first, rapidly occupying niches before declining from resource depletion or competition. Their decline created opportunities for members of *Cyanophyceae* and *Alphaproteobacteria* (which tend to exploit more labile resources) to establish themselves in the following phase of succession, particularly at stations N01 and N08 (Fig. 3C, E). This pattern aligns with niche differentiation theory [53, 54], where different taxa occupy distinct ecological roles over time, effectively partitioning the available resources. The shift from *Gammaproteobacteria* to *Alphaproteobacteria* and *Cyanophyceae* representatices follows a secondary succession pattern, where species with higher competitive abilities replace pioneer taxa once the initial conditions are modified [52]. *Alphaproteobacteria* members were the most persistent group in our study, being present throughout the entire incubation period at certain stations and suggesting an adaptive advantage in oligotrophic conditions. Their sustained presence at station N08, where they colonized both alginate and agarose particles, highlights their resilience in nutrient-poor environments, likely due to efficient nutrient uptake systems [55]. This persistence contrasts with a decline in *Gammaproteobacteria* ASVs, highlighting a competition-colonization trade-off [56]. Similarly, “*Betaproteobacteria”* members exhibited delayed but notable increases in abundance later in the incubation period, suggesting a more opportunistic strategy. At stations N08, N12, and N16, “*Betaproteobacteria*” peaked around 36 h, exemplifying secondary succession dynamics where organisms colonize pre-conditioned substrates [57] (Fig. 3C, D, E, G, H). This delayed colonization strategy allows “*Betaproteobacteria”* members to avoid competition with faster colonizers and simultaneously exploit environments modified by earlier colonizers, demonstrating temporal niche partitioning [58]. However, members of “*Betaproteobacteria”* are expected to face competition from other non-diazotrophic bacteria, which could also be competing for available resources. Finally, *Cyanophyceae* representatives demonstrated variable colonization dynamics influenced by abiotic factors like light intensity. In the Arctic summer, high and continuous light exposure can induce oxidative stress in cyanobacteria, potentially leading to a shift towards mixotrophy to meet energy needs [23, 24, 59, 60]. This adaptation provides *Cyanophyceae* members with a competitive advantage in environments where other non-phototrophic diazotrophs are less suited to stable light and nutrient conditions.

The colonization patterns of *Gammaproteobacteria*, *Alpha*proteobacteria, “*Betaproteobacteria”*, and *Cyanophyceae* members reveal how biotic and abiotic factors shape diazotrophic communities. This complexity reflects the diverse adaptive strategies that microbial taxa employ to thrive in nitrogen-poor, carbon-rich, and environmentally dynamic marine ecosystems.

### Colonization patterns of diazotrophs vs. non-diazotrophs

Diazotrophs are not the only colonizers of organic particles in the marine environment. To better understand particle colonization and niche differentiation patterns, we compared *nifH* and 16S rRNA gene amplicon datasets to assess the distinct colonization dynamics of diazotrophic and non-diazotrophic microbial communities, respectively. In the Barents Sea, the high and increasing abundance of brown algae [61], particularly kelp, likely plays a key role in structuring these microbial communities and influencing carbon cycling pathways. Recent studies indicate that kelp biomass is not only increasing but is also distributed closer to the surface [62], enhancing its ecological and biogeochemical impact. Kelp biomass can reach up to 20 kg m^−2^ during the summer along the coasts of Svalbard [63], providing a substantial source of organic matter including alginate, a polysaccharide predominant in brown algae. Organic compounds derived from brown algae such as alginate support microbial communities in Arctic regions, significantly contributing to carbon cycling through biodegradation [48, 62, 64]. In contrast, red algae which are also present in this region contribute to the diversity of polysaccharides in seawater, For exemple agarose, but with much lower biomass (approximately 0.5 kg m^−2^; [63, 65]). This disparity in biomass between brown and red algae suggests that alginate is likely a more accessible and influential carbon source in the Barents Sea. The observed preference for alginate across most diazotrophic groups may thus reflect the ecological availability and importance of this compound.

Diazotrophs preferentially colonized alginate over agarose (Fig. 4; Table S1), indicating that alginate-rich brown algae leachates are important substrates for N_2_ fixation in this region. In contrast, non-diazotrophs exhibited a greater versatility in their carbon source preferences, colonizing both alginate and agarose with similar effectiveness. Alternatively, non-diazotrophs were more versatile in their carbon source preferences (Fig. 4; Table S2). This versatility likely reflects the diversity of taxa captured by 16S rRNA gene amplicon sequencing, which includes bacteria with a broad range of metabolic capabilities. The bulk prokaryotic community harbored diverse metabolic capabilities, accessing both complex polysaccharides and simple carbon substrates beyond diazotrophs’ reach [47, 66]. Additionally, non-diazotrophs are thought to rely on N_2_ fixed by diazotrophs reducing their need to invest energy in nitrogen acquisition processes [67]. The distinct colonization patterns between diazotrophs and non-diazotrophs demonstrate resource partitioning at both spatial and temporal scales [68]. This partitioning follows metacommunity theory principles, where local community assembly is influenced by both environmental filtering and species interactions [69].

The competition for resources between diazotrophs and non-diazotrophs is a crucial factor in understanding the cycling of nutrients in the increasingly nitrogen-limited Arctic. Given the substantial energy requirements for N_2_ fixation (16 ATP per N_2_ molecule; [70]), diazotrophs need to colonize carbon-rich particles to secure a stable energy supply [22]. Non-diazotrophs, however, may benefit indirectly from the degradation of complex organic matter by diazotrophs or from the release of freshly fixed N_2_ [71] without needing to directly colonize and degrade these particles. Here we observed that NCDs have a propensity to colonize alginate-rich particles faster than non-diazotrophs (Fig. 3; Table S1). In natural marine snow, non-diazotrophic bacteria initially colonize nitrogen-rich particles, depleting the nitrogen, and creating anoxic cores that diazotrophs later colonize to access carbon-rich environments needed for N_2_ fixation [31]. Furthermore, studies have shown that many microorganisms including non-diazotrophic bacteria exhibit chemotactic responses to microgradients of bioavailable nitrogen compounds such as nitrite, ammonium, and nitrate [68]. In our study, however, the artificial particles used did not contain nitrogen [72, 73], which likely discouraged an initial colonization by non-diazotrophs. Diazotrophs may have thus directly colonized these nitrogen-poor carbon-rich particles, using the available carbon to meet their energy demands for subsequent N_2_ fixation [22]. In nitrogen-limited Arctic environments, diazotrophs’ early colonization of carbon-rich particles may contribute to nitrogen cycling when bioavailable nitrogen is scarce.

Even though our artificial particles were nitrogen-free, this composition mirrors late-stage marine particles where preferential nitrogen remineralization leads to higher C:N ratio. As marine snow sinks and ages, its C:N ratio increases from 7.1 to >8.5 due to microbial nitrogen consumption [74], creating nitrogen-depleted microenvironments that potentially favor N_2_-fixing diazotrophs [75]. This aligns with a recent study [76], which found that high C:N ratios in marine particles correlate with increased diazotrophic activity, suggesting carbon availability promotes N_2_ fixation.

### NCDs prefer colonizing alginate over agarose

**Figure 6:**
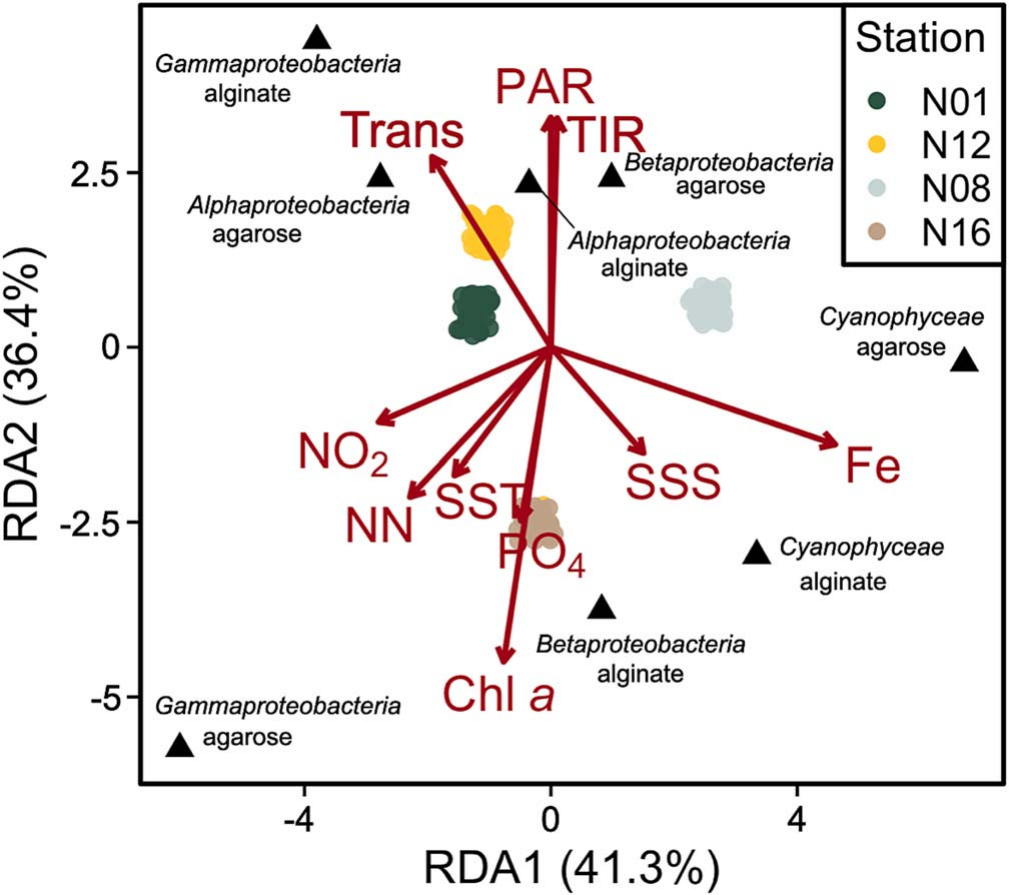
Redundancy analysis (RDA) triplot representing the distribution of individual samples as a function of preferences for carbon sources (agarose and alginate) and environmental variables measured at the sampling stations. The dots indicate the samples, with specific colors for each station. The red vectors show the gradients of the environmental variables influencing carbon preferences, their length and direction illustrating the strength and direction of the effect on the groups of microorganisms. The percentage values indicate the inertia explained by the first two axes of the RDA.

Diazotrophic *Gammaproteobacteria* members were the best competitors for alginate. This class is known for its diverse metabolic capabilities, being able to utilize a wide range of organic substrates including alginate [47]. The preference for alginate at all stations except N16 highlights the significant role of *Gammaproteobacteria* representatives in polysaccharide degradation in the Barents Sea. This observation aligns with habitat selection theory [77], where organisms actively choose environments that maximize their fitness. The preference for alginate suggests resource specialization, a key mechanism enabling species coexistence in marine systems [78]. In the Arctic, labile organic carbon is often limited outside the brief June–September productivity bloom window [5, 79]. At station N16, near the Negribreen glacier (Fig. 2A), glacial melt, run-off, and possible localized upwelling could increase organic matter concentrations, particularly algal-derived alginates [80]. For diazotrophs, especially heterotrophic ones, colonizing organic-rich particles can be crucial for meeting their metabolic needs in nutrient-poor environments [42], likely explaining their faster colonization of these particles. At station N16, however, Gammaproteobacteria members showed a clear preference for agarose instead of alginate. This station had the highest Chl *a* concentration observed during our cruise indicating high organic matter loading in the water column, as also supported by the lower PAR values (Fig. 2F). A redundancy analysis (RDA) shows that the agarose preference of *Gammaproteobacteria* representatives aligns with Chl *a* vector, suggesting Chl *a* concentrations influence this preference (Fig. 6). This observation is consistent with a previous study [78], which demonstrated the rapid and targeted succession of *Gammaproteobacteria* members in response to algal decay, highlighting their opportunistic capacity to exploit algal-derived organic matter.

High natural alginate levels from brown algae at station N16 may saturate the chemotactic response of *Gammaproteobacteria* representatives, reducing their attraction to artificial alginate particles. In contrast, the lower environmental concentrations of agarose may have enhanced the sensitivity of these bacteria to agarose particles [81]. Bacteria can modify their chemotactic responses based on the prevailing concentrations of chemical cues in their immediate environment [82]. High ambient concentrations of a substrate can lead to adaptation and reduced sensitivity, as described by the Weber–Fechner law, and conversely, lower levels may enhance sensitivity [83]. This dynamic is expected to help *Gammaproteobacteria* members to disperse and colonize new areas quickly, even when food is abundant locally [84]. At station N16, preference for agarose likely reflects chemotactic adaptation, in contrast to other stations where substrate specialization explains the general preference for alginate.

The lack of a clear carbon source preference in *Alphaproteobacteria* representatives may suggest their limited ability to degrade complex polysaccharides like alginate and agarose. Instead, they may rely on simpler organic compounds, reflecting their adaptation to oligotrophic conditions and specialization in low-molecular-weight substrates [85]. This aligns with the “Black Queen Hypothesis”, which proposes that some microorganisms abandon costly functions and rely on “public goods” produced by others [86], with recent studies suggesting that such metabolic interdependence structures microbial communities and drives their co-evolution [87]. Field observations support this: certain *Alphaproteobacteria* taxa lack enzymes to degrade complex organic matter and instead depend on metabolites from other organisms [88]. In contrast, “*Betaproteobacteria”* exhibit diverse metabolic capabilities [89], which stands to explain their lack of a pronounced preference for a specific carbon source. This metabolic diversity may stem from genetic variability among populations at different stations. Diazotrophic cyanobacteria showed either a significant preference for alginate particles or no substrate preference across stations (Table S2). At stations N08 and N16, they exhibited higher relative abundances on alginate (42.5% and 56.8% respectively, Fig. 4) compared to agarose particles (26.2% and 37.1% respectively, Fig. 4), yet showed comparable colonization patterns on both substrates at stations N01 and N12 (Fig. 3). This observation suggests that cyanobacteria can effectively establish themselves on both particle types through different ecological strategies. On alginate particles, where they showed significant preference at two out of four stations, diazotrophic cyanobacteria can directly degrade this substrate. However, on agarose particles, they likely rely on cross-feeding relationships with heterotrophic bacteria capable of breaking down the complex polysaccharide [75, 76]. Such substrate flexibility is characteristic of marine microbial communities, where metabolic networking enables efficient utilization of diverse organic particles [55, 77]. In marine environments, these interactions enable efficient utilization of complex substrates by microbial consortia on organic particles. Additionally, the dFe concentration at the different stations (Fig. 2G) supports cyanobacterial growth and nitrogen fixation, enhancing their ability to utilize degradation products from both particle types [90, 91]. The temporal dynamics of colonization further illuminate this pattern: whereas *Gammaproteobacteria* members reached full colonization of alginate particles in 72 h, *Cyanophyceae* representatives achieved similar relative abundances on both substrates (Fig. 3), suggesting their ability to establish viable populations regardless of substrate composition [53, 92]. The RDA reveals substrate-specific responses, with iron availability particularly influencing cyanobacterial colonization on alginate particles. In comparison their presence on agarose particles shows weaker correlations with the measured environmental parameters (Fig. 6).

## Conclusions

Our study details diazotroph particle colonization in the Arctic Ocean, highlighting selective substrate preferences and roles in nitrogen cycling. We observed that *Gammaproteobacteria* representatives, a dominant group of NCDs, preferentially colonize alginate particles with abundant polysaccharides from brown algae, becoming more prevalent with Arctic warming. This preference suggests a close ecological connection between these diazotrophs and algal-derived organic matter. The distinct colonization patterns between diazotrophic and non-diazotrophic bacteria indicate niche partitioning within the microbial community. Typically, non-diazotrophs colonize first, attracted by bioavailable nitrogen, which they deplete over time leaving nitrogen-poor particles for diazotrophs that can fix N_2_ to meet their needs. In our experiment, nitrogen-free carbon-rich particles may have favored colonization by diazotrophs, which have their own means to obtain nitrogen. Conversely, non-diazotrophs reliant on external nitrogen sources were less inclined to colonize nitrogen-free particles. This delayed colonization by diazotrophs suggests they play a secondary but essential role in nitrogen cycling, particularly after nitrogen depletion. When diazotrophs showed a strong affinity for alginate, likely due to their high energy requirements for N_2_ fixation, non-diazotrophs exhibited broader versatility, with no clear preference between alginate and agarose, suggesting different metabolic strategies.

Arctic warming will increase algal blooms, runoff, and glacial melt, increasing iron and alginate levels, favoring particle-associated diazotrophs and N_2_ fixation. This shift is anticipated to significantly impact Arctic biogeochemical cycling, nutrient availability, and primary production in nitrogen-limited waters. Understanding these diazotroph-particle interactions is essential for predicting the Arctic Ocean’s response to climate change. Parallel N_2_ fixation measurements were conducted during this cruise using ^15^N_2_ tracer incubations, future studies combining ^15^N_2_ labeled particles with single-cell mass spectrometry analyses (i.e., nanoSIMS) would enable direct visualization of nitrogen incorporation at the cellular level. Additionally, further research should investigate metabolic interactions between and within diazotroph and non-diazotroph groups, as their selective colonization of algal particles may reshape Arctic nitrogen cycling and ecosystem dynamics.

## Supplementary Material

FigureS1_wraf098

FigureS2_wraf098

FigureS3_wraf098

FigureS4_wraf098

FigureS5_wraf098

FigureS6_wraf098

FigureS7_wraf098

FigureS8_wraf098

TableS1_wraf098

TableS2_wraf098

TableS3_wraf098

TableS4_wraf098

TableS5_wraf098

TableS6_wraf098

TableS7_wraf098

TableS8_wraf098

Supplementary_material_ISMEJ-D-25-00082_wraf098

## Data Availability

The *nifH* and 16S rRNA gene sequences generated during this study have been deposited in the NCBI Sequence Read Archive (SRA) under accession numbers PRJNA1188574 and PRJNA1189317, respectively. All other data generated or analyzed during this study are included in this published article and its supplementary information files. The device specifications and 3D printing files for the In-Situ Chemotaxis Assay are available at: https://github.com/OceanBridges/In-Situ-Chemotaxis-Assay-adapted-version.
