# Supplementary figures and images for: Dynamics of diazotroph particle colonization in the Arctic Ocean
Source: ISME J. 2025 May 20;19(1):wraf098. doi: 10.1093/ismejo/wraf098 (PMC12202154; doi:10.1093/ismejo/wraf098)

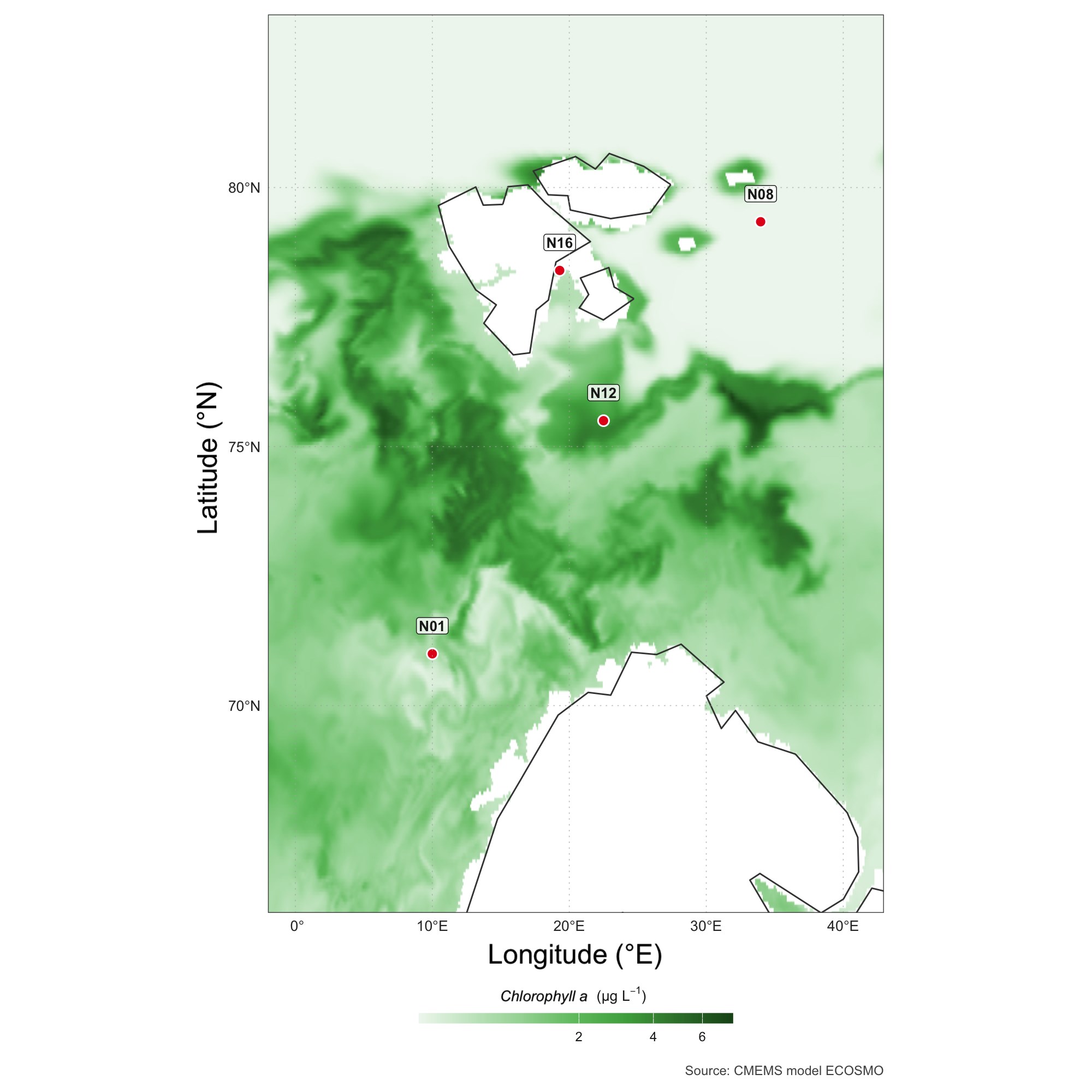

Supplement: FigureS1_wraf098 [file figures1_wraf098.jpeg]

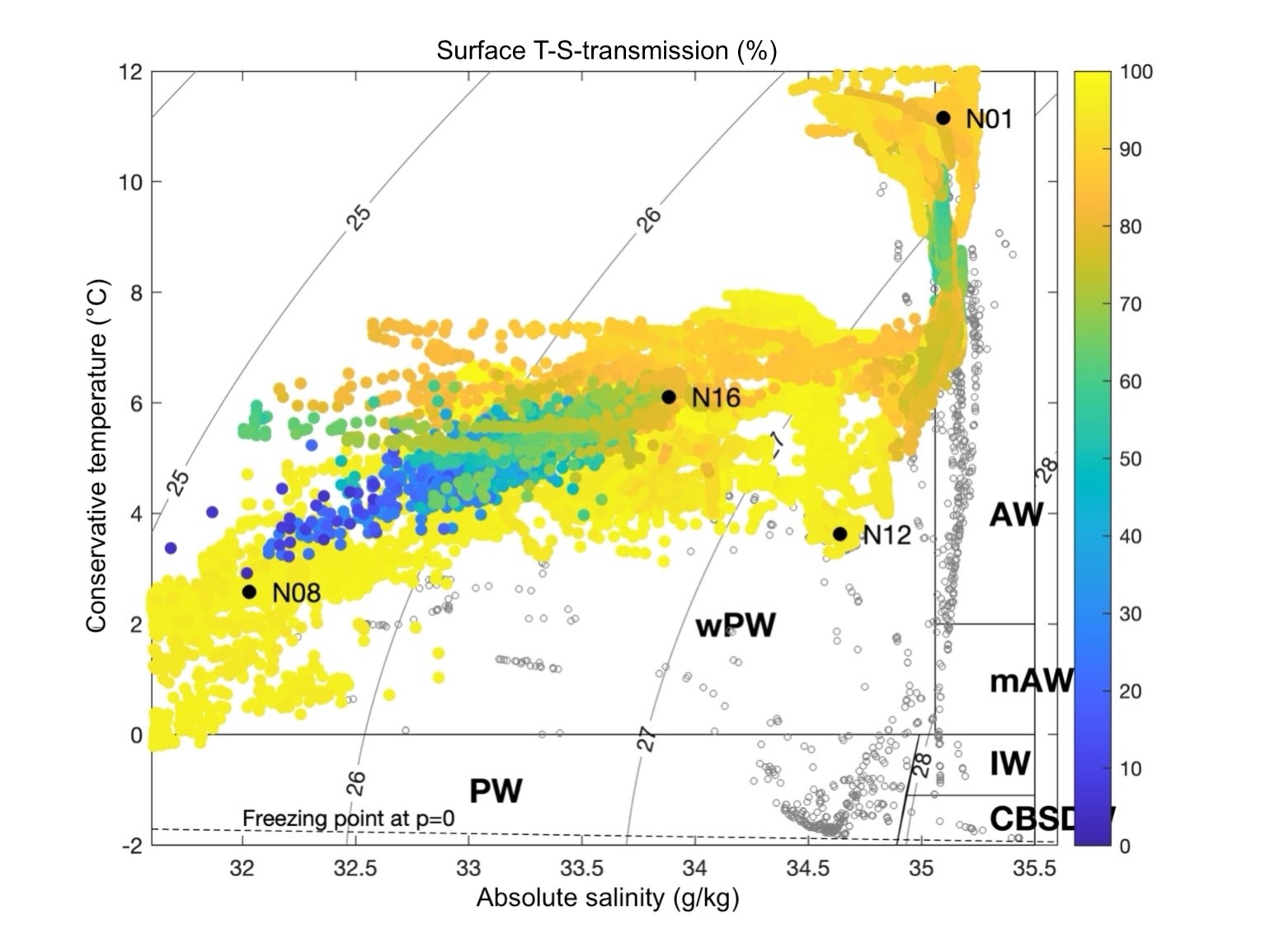

Supplement: FigureS2_wraf098 [file figures2_wraf098.jpeg]

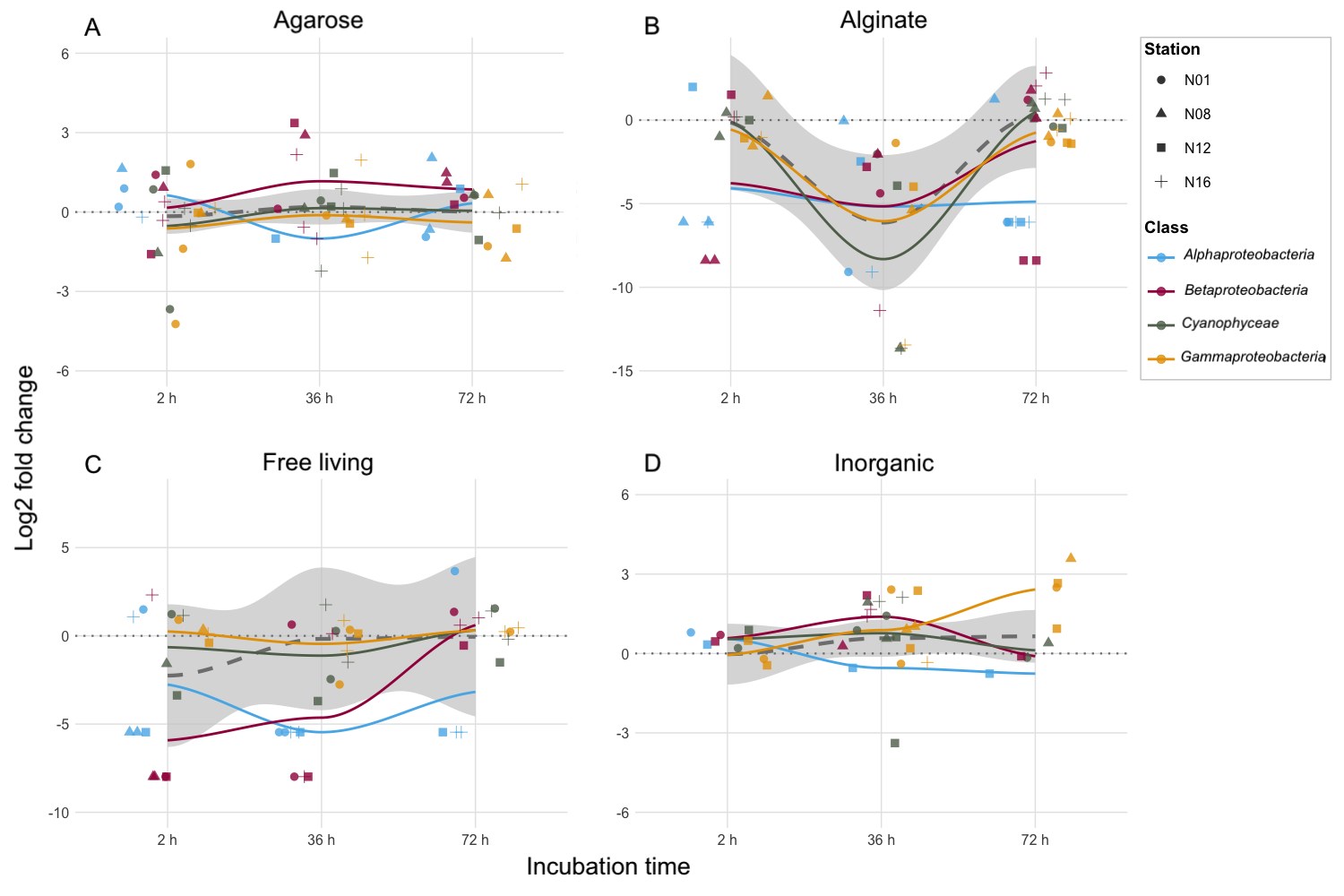

Supplement: FigureS3_wraf098 [file figures3_wraf098.jpeg]

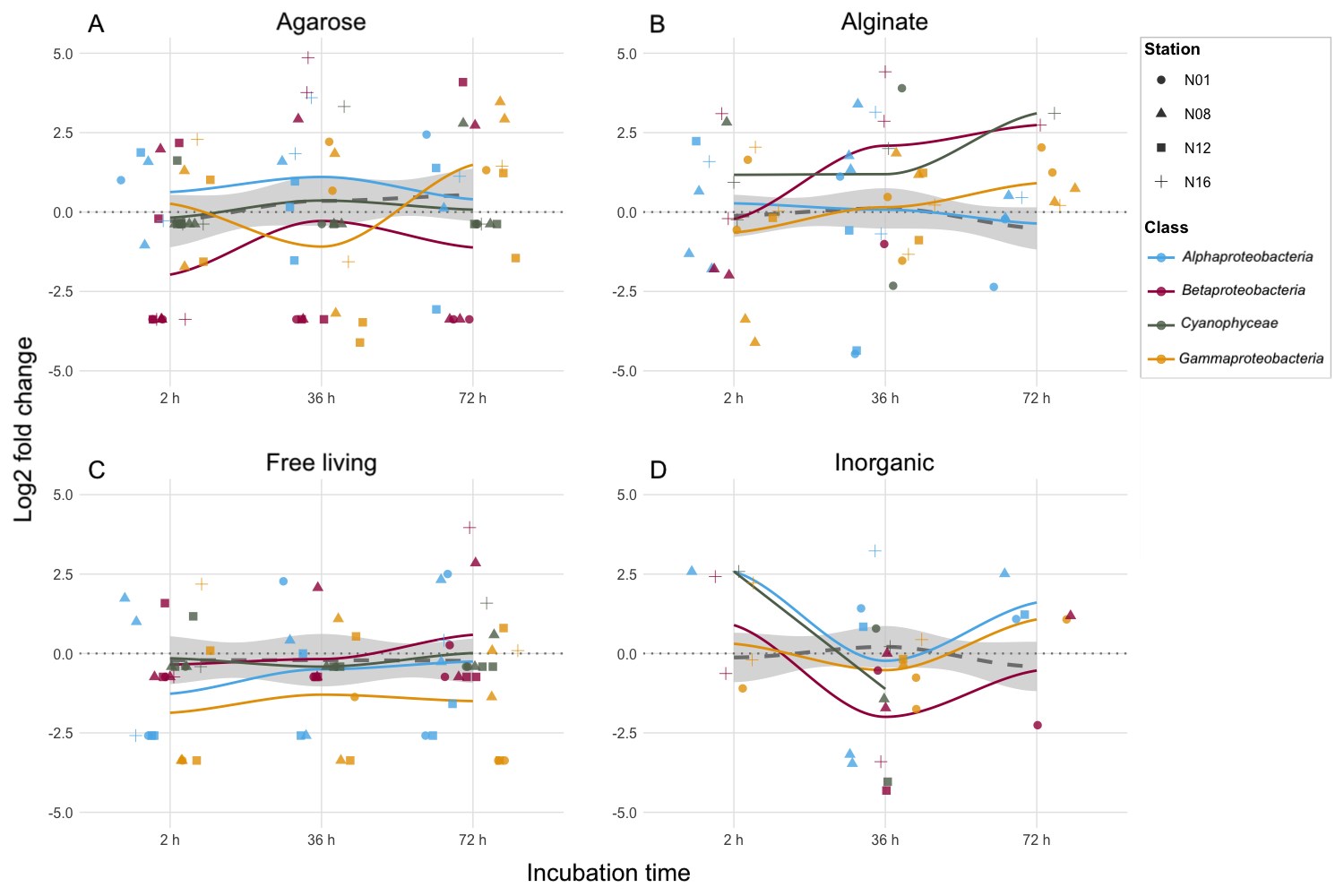

Supplement: FigureS4_wraf098 [file figures4_wraf098.jpeg]

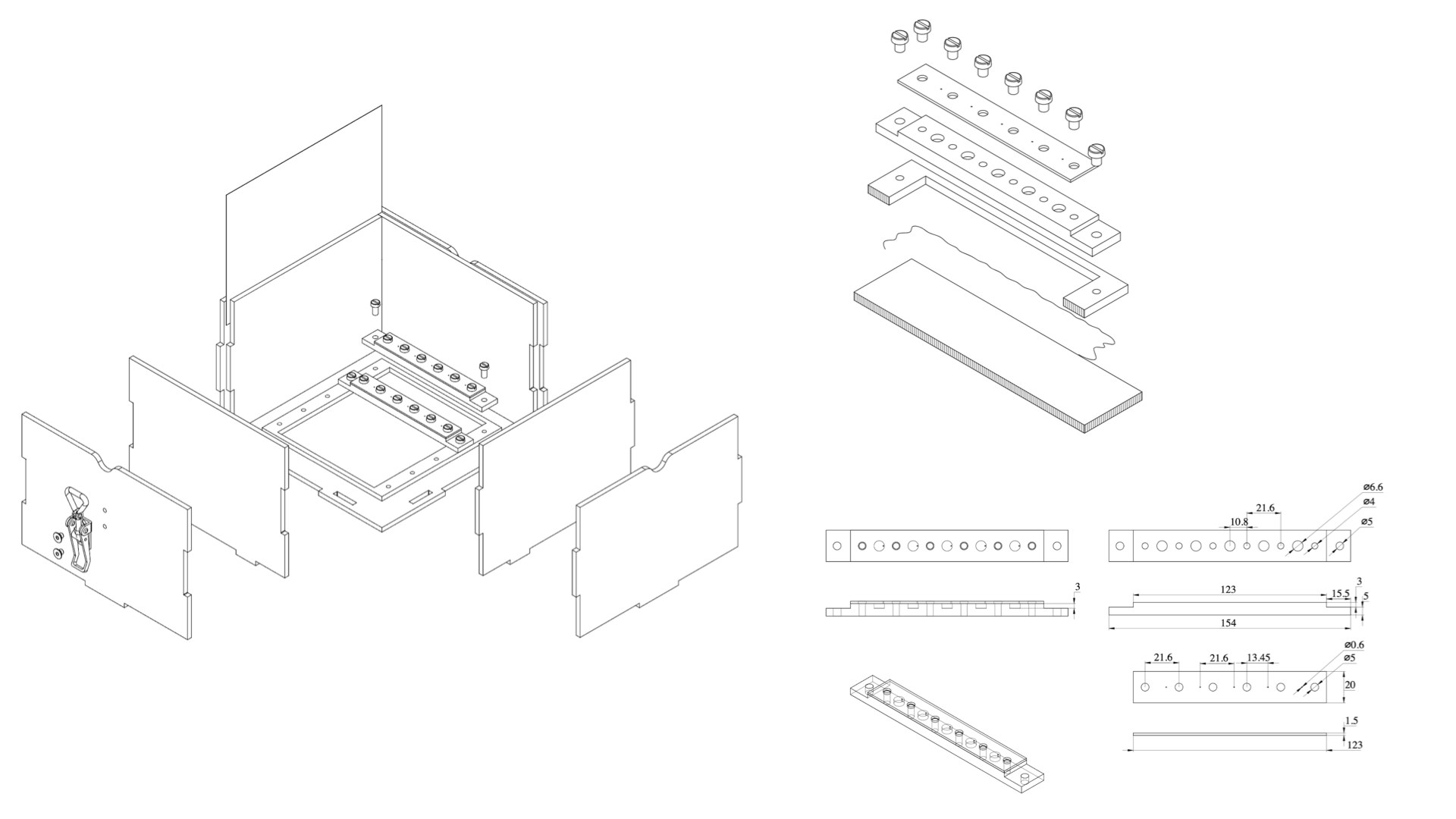

Supplement: FigureS5_wraf098 [file figures5_wraf098.jpeg]

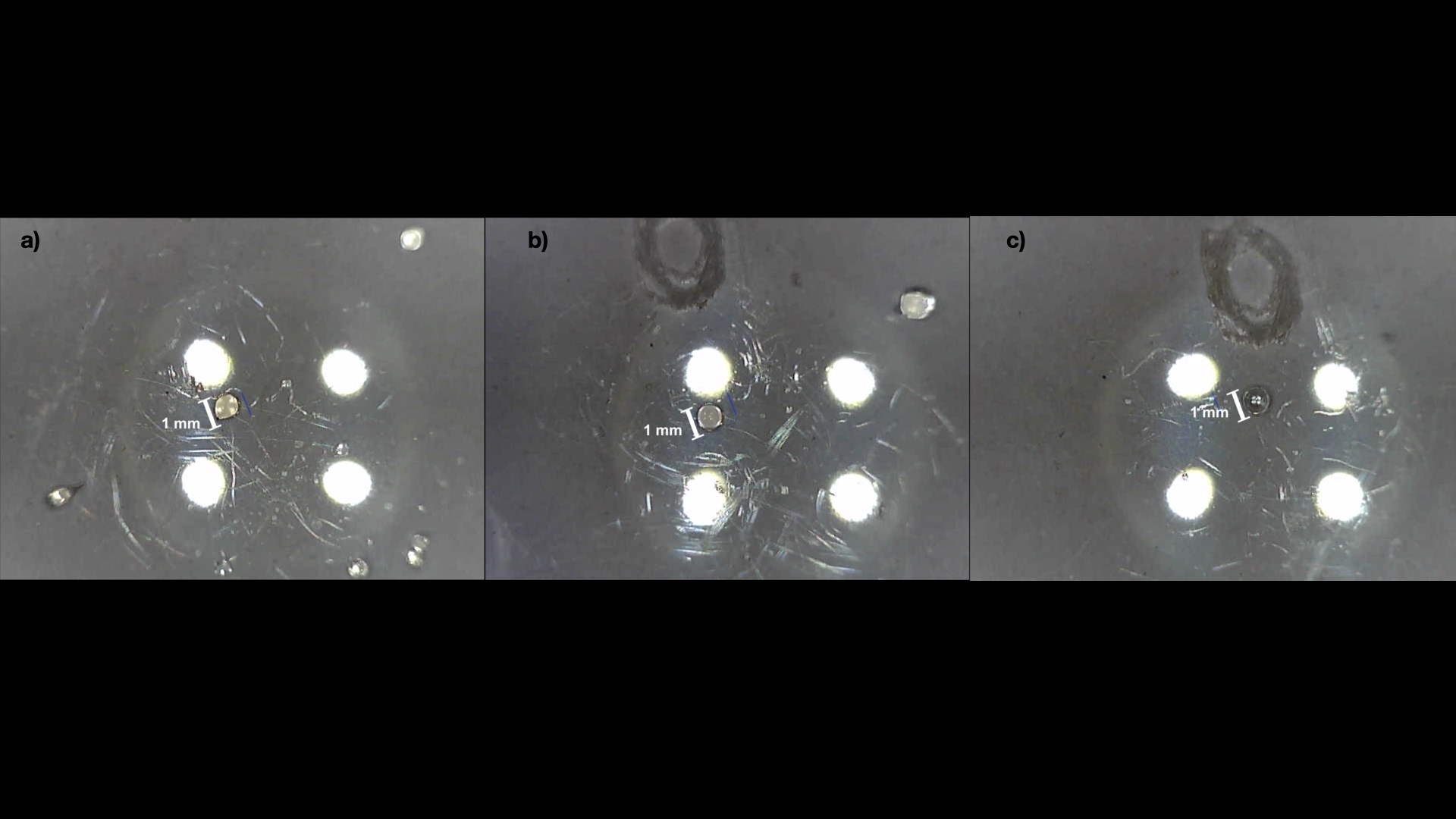

Supplement: FigureS6_wraf098 [file figures6_wraf098.jpeg]

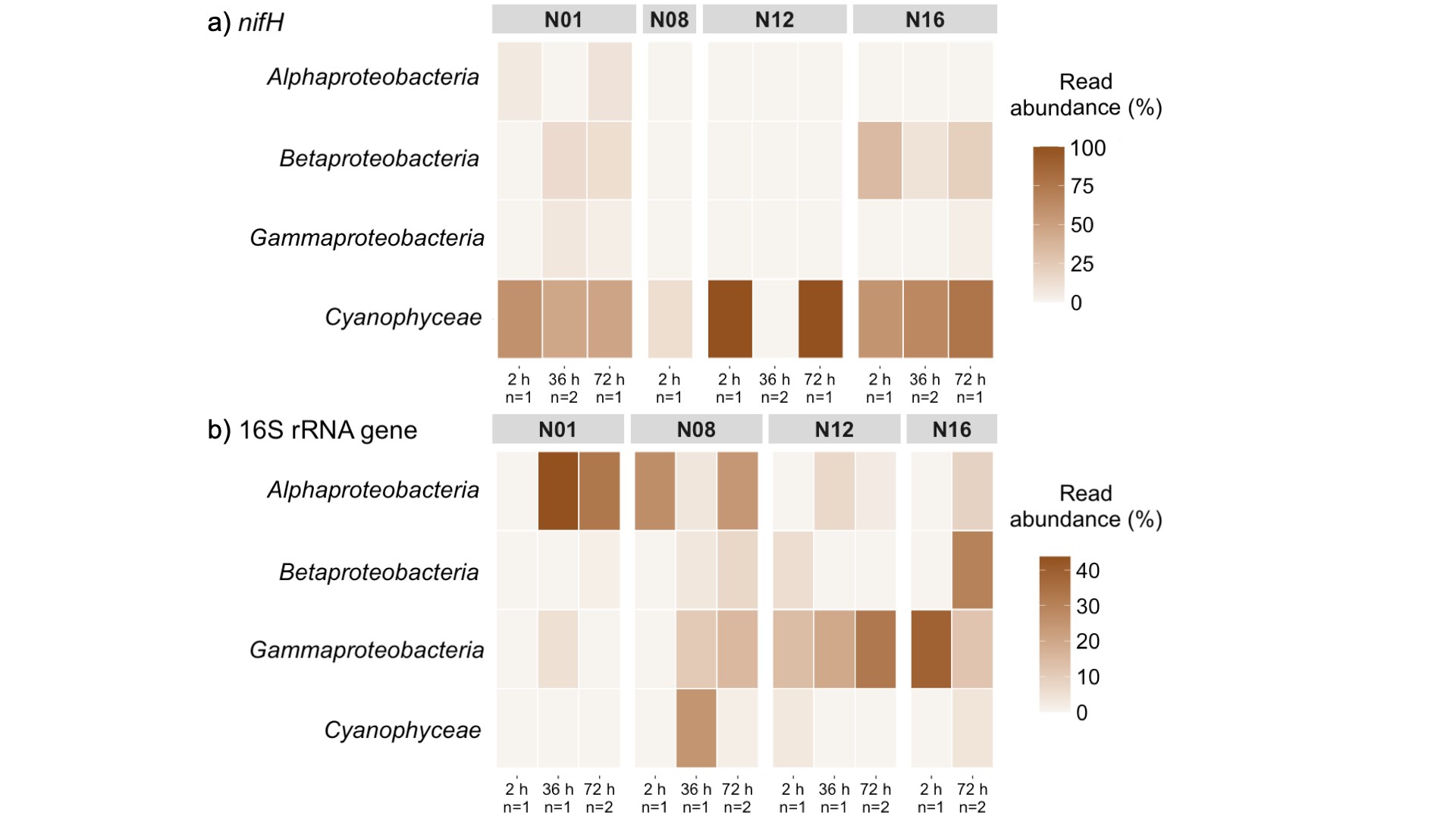

Supplement: FigureS7_wraf098 [file figures7_wraf098.jpeg]

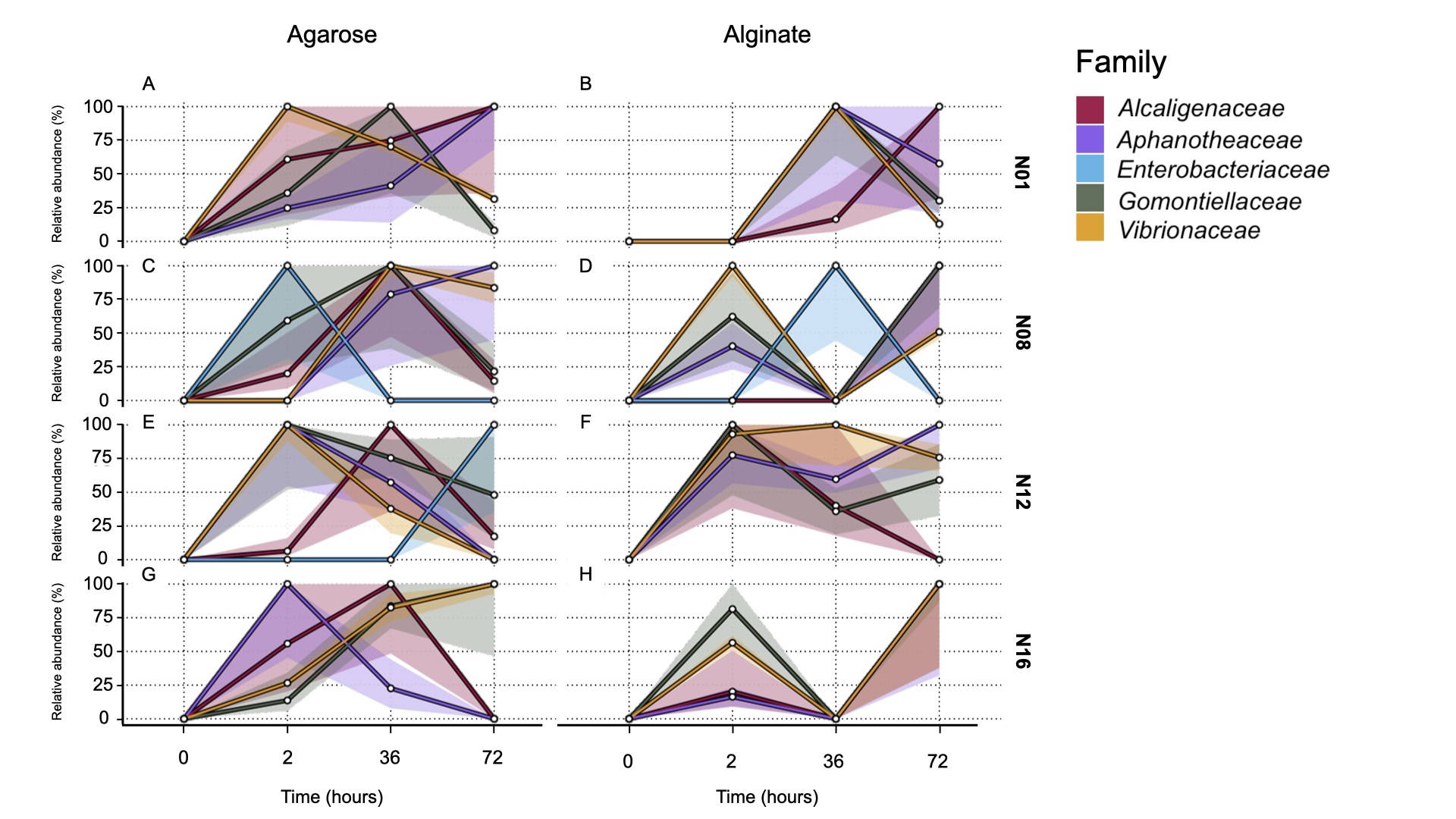

Supplement: FigureS8_wraf098 [file figures8_wraf098.jpeg]
