# Supplementary material for: Dynamics of diazotroph particle colonization in the Arctic Ocean
Source: ISME J. 2025 May 20;19(1):wraf098. doi: 10.1093/ismejo/wraf098 (PMC12202154; doi:10.1093/ismejo/wraf098)
Supplement: Supplementary_material_ISMEJ-D-25-00082_wraf098 [file supplementary_material_ismej-d-25-00082_wraf098.pdf]

Supplementary information

Dynamics of diazotroph particle colonization in the Arctic Ocean

Arthur Coët[1,2], Cécile Carpaneto Bastos[1,2], Mathias Lechelon[3], Ruth Hawley[4], Oliver Flanagan[4], Maeve C. Lohan[4], Pierre Ronceray[2], Joanne E. Hopkins[5], Claire Mahaffey[4], Mar Benavides[1,2,6*]

(1) Aix Marseille Univ, Université de Toulon, CNRS, IRD, MIO UM 110, Marseille, France

(2) Aix Marseille Univ, CNRS, CINAM, Turing Center for Living Systems, Marseille, France

(3) Aix Marseille Univ, CNRS, INSERM, MEP Centuri, Turing Center for Living Systems, Marseille, France

(4) Department of Earth, Ocean, and Ecological Sciences, University of Liverpool, Liverpool, UK

(5) National Oceanography Centre, Liverpool, L3 5DA, UK

(6) National Oceanography Centre, European Way, Southampton, SO14 3ZH, UK

*Corresponding author : Mar Benavides, National Oceanography Centre, European Way, Southampton, SO14 3ZH, UK

Running title : Arctic particle-attached diazotrophs

Keywords : Nitrogen fixation, Organic matter, Chemotaxis, Barents Sea

arthur.coet@mio.osupytheas.fr, cecile.carpaneto-bastos@mio.osupytheas.fr, mathias.LECHELON@univ-amu.fr, rh3g22@soton.ac.uk, M.Lohan@soton.ac.uk, pierre.RONCERAY@univ-amu.fr, jeh200@noc.ac.uk, mahaffey@liverpool.ac.uk, marben@noc.ac.uk

Supplementary Methods

Sampling

Sampling was conducted during the N-ARC DY167 cruise on board the RRS *Discovery* between 15th July and 6th August 2023. At each station, surface seawater samples were collected using a trace metal clean tow-fish positioned at approximately 5 m below the surface (adjustable to 7 m) and filtered through 10 μm polycarbonate filters to remove large particles, phytoplankton cells, and zooplankton. Nutrient concentrations (nitrate, nitrite, ammonium, phosphate, and silicate) were measured from seawater samples using a Bran and Luebbe QuAAtro 5-Channel Nutrient Auto-analyzer with detection limits of 0.1 μM for nitrate plus nitrite and silicate, and 0.05 μM for phosphate, ammonium and nitrite. Samples for dissolved iron (dFe) measurements were taken from 1–2 m depth, filtered through 0.2 μm Sartorius filters (Göttingen, Germany), and acidified to 0.012 M using ultra-pure HCl. The concentration of dFe was determined using the iron chemiluminescence detection following the method of Kunde et al., 2019, which also outlines the tow-fish sampling protocols. Particulate trace metal samples (iron, manganese, aluminum) were collected from OTE bottles onto acid-cleaned 25 mm Supor® polyethersulfone membrane disc filters (0.45 μm pore size; Pall, NY, USA). These filters were stored frozen at -20°C until shore-based analysis. The filtered volumes varied between 1–5 L depending on the sample depth, and this was accounted for in the calculations. Particulate trace metals were analyzed using ICP-MS following the procedures outlined in the Sampling and Sample-handling Protocols for GEOTRACES Cruises, Version 3.0, 2017. Sea surface temperature was recorded using two sensors on the TSG system of TechSAS, measuring water at a depth of 5.5 m with a sampling frequency of 1 Hz. Data were despiked using a 3-point median filter and subsequently averaged over 1- and 10-minute intervals, expressed in degrees Celsius. Sea surface salinity (SSS) was obtained from the same TSG system at 1 Hz and

processed similarly with a 3-point median filter. Salinity data were calibrated against underway samples analyzed with AUTOSAL and standard seawater, correcting for sensor drift via robust linear fitting in MATLAB. Chlorophyll a fluorescence was measured using the surfmet system at 1 Hz, despiked with a 5-point median filter, and averaged to 1-minute intervals. Fluorescence values were converted to chlorophyll concentrations using a calibrated linear equation based on onboard sample sets.

Single artificial particle DNA extraction and quantification

DNA was extracted from single artificial particles using the Agencourt DNAdvance kit, 96 well (Beckman Coulter, Brea, California, USA) inspired by Pontrelli et al. (2022)(1) which successfully demonstrated this kit's effectiveness for single-particle DNA extractions. The manufacturer's protocol was modified by the substitution of 2 mL Eppendorf tubes instead of standard 96-well microplates. For magnetic separation, a magnetic rack specifically designed for Eppendorf tubes, manufactured by Sergi Lab (Seattle, Washington, USA), was used instead of the conventional magnetic rack, which provided a better fit for our modified setup. In addition, the volume of the elution buffer was adjusted to 30 µL to increase the concentration of the DNA obtained. After DNA was extracted from the samples, the concentration of the purified DNA was quantified using the ThermoFisher Scientific Quant-iT™ PicoGreen™ dsDNA Assay (Waltham, MA, USA).

Amplicon sequencing

The *nifH* gene was amplified using a nested PCR approach. Primers *nifH*3 (5'-ATRTTRTTNGCNGCRTA-3') and *nifH*4 (5'-TTYTAYGGNAARGGNGG-3') were used initially, with a master mix containing 5 µL MyTaq Red PCR Buffer, 0.5 µL of each primer, 1.25 µL $MgCl_2$, 0.5 µL bovine serum albumin, 0.25 µL Taq DNA Polymerase, and PCR-grade water to a

total of 25 µL. The second PCR used primers *nifH*1 (5'-TGYGAYCCNAARGCNGA-3') and *nifH*2 (5'-ADNGCCATCATYTCNCC-3'), with 1 µL of the first PCR product as template and an identical master mix. The PCR program was identical for both rounds: initial denaturation at 94°C for 2 min; followed by 30 cycles of 94°C for 1 min, 54°C for 1 min, and 72°C for 1 min 30 sec; with a final elongation at 72°C for 7 min. TaqMan™ Universal PCR Master Mix (2x5mL, Fisher, Hampton, New Hampshire, United States) was used. To increase the reliability and yield of our results, we performed each second-round PCR in triplicate. After amplification, we subjected each PCR product to electrophoresis on a 1.5% agarose gel. We included DNA extracted from *Vibrio diazotrophicus* as a positive control and PCR-grade water as a negative control to ensure the validity of our PCR results. Bands indicating successful amplification were visualized under UV light, excised from the gel using a sterile scalpel, and then pooled in a single tube. To obtain clean and concentrated DNA suitable for subsequent analyses, these gel-extracted DNA fragments were purified using the GeneClean Turbo Kit (MP BioMedical, Santa Ana, CA, USA) according to the manufacturer's instructions. For *16S* rRNA gene analysis, extracted DNA samples were sent to GeneXmap (Marseille, France) for amplification and quantification. Subsequent sequencing of 2 x 300 bp paired-end reads, providing comprehensive coverage and depth for accurate sequence analysis. A nested PCR approach was used, beginning with amplification of the V1-V9 region in the first PCR, followed by targeting the V3-V4 region in the subsequent PCR, to maximize amplification of the region of interest. The primers used were: 16S rRNA gene Amplicon PCR Forward Primer (5' TCGTCGGCAGCGTCAGATGTGTATAAGAGACAGCCTACGGGNGGCWGCAG) and 16S rRNA gene Amplicon PCR Reverse Primer (5' GTCTCGTGGGCTCGGAGATGTGTATAAGAGACAGGACTACHVGGGTATCTAATCC). Sequencing was performed with 2 x 300 bp paired-end reads, providing comprehensive coverage and depth for accurate sequence analysis.

Bioinformatics

The R environment DADA2 pipeline (v1.31.0) was used to process the sequencing data (2). Using the filterAndTrim tool, raw sequence reads were first quality filtered and trimmed with varied parameters for 16S rRNA and *nifH* gene sequences.

The 16S rRNA gene sequences were subjected to the following modifications: reads with ambiguous nucleotides were discarded (maxN=0), reads with forward and reverse reads were truncated to 250 base pairs (truncLen=c(250,250)), and reads exceeding the expected error rates of 2 for forward reads and 2 for reverse reads were removed (maxEE=c(2,2)). A minimum quality score of two (truncQ=2) was also used to filter the reads, and sequences matching the phiX genome were eliminated (rm.phix=TRUE). Multiple threads were used to carry out the filtering operation simultaneously (multithread=TRUE). For genus-level analyses of 16S rRNA gene data, we aggregated OTUs using the tax_glom function in phyloseq. Community structure differences were assessed using non-parametric permutational multivariate analysis of variance (PERMANOVA, adonis2 function) with Bray-Curtis distances. Alpha diversity (Shannon index) differences between substrate types were tested using Kruskal-Wallis rank sum tests. Individual genus preferences for specific substrates were evaluated using Kruskal-Wallis tests on relative abundances, with $p < 0.05$ considered significant.

The forward and reverse reads for the *nifH* gene sequences were each truncated to 220 base pairs and 160 base pairs, respectively (truncLen=c(220,160)). The expected error rate for both forward and reverse reads was set to 2 (maxEE=c(2,2)), and the maximum number of ambiguous nucleotides permitted was set to 0 (maxN=0). TruncQ=19, a stricter quality score cutoff, was used. PhiX sequences were eliminated as well (rm.phix=TRUE), and multithreading was turned off on Windows platforms in order to compress and execute the filtering process in

parallel (compress=TRUE, multithread=TRUE). The *nifH* and 16S rRNA gene sequences have been deposited in the NCBI Sequence Read Archive (SRA) database under the accession number [PRJNA1188574] and [PRJNA1189317] respectively.

For the taxonomic assignment, the 16S rRNA gene sequences were taxonomically assigned using the Silva reference database (v138.1). The training set file silva_nr99_v138.1_train_set.fa.g was employed for the initial classification, while species-level assignments were refined using the silva_species_assignment_v138.1.fa.g file. For *nifH* gene sequences, taxonomic annotation was conducted using the DADA2 formatted *nifH* database version 2.0.5, from M. A. Moynihan & C. Furbo Reeder 2023, provided in the Fasta format. These steps ensured accurate taxonomic classification of the microbial communities present in the samples, facilitating further ecological and functional analyses. A 80% threshold for the bootstrap was used, to ensure a good taxonomic affiliation of the different taxonomic groups. All bioinformatics and statistical analyses were performed using the R programming environment (4.4.1 ; 2024-06-14). Several R packages were used to process, analyze, and visualize the microbiome data. Sequencing data were first processed using the dada2 package (2), which was used for quality filtering, denoising, and construction of the amplicon sequence variant (ASV) table. Rcpp (3) was used to increase the computational efficiency of these processes. Subsequent data manipulation and statistical analyses were performed using the tidyverse suite of packages (4), including dplyr for data manipulation, tidyr for data tidying, and tibble for data frame handling. Microbiome data were further analyzed using the phyloseq package (5)to handle and visualize phylogenetic sequencing data. vegan (6) was used for ecological diversity analysis, and microbiome and microViz (7)were used for advanced microbiome analysis and visualization. To create visual representations of the data, we used ggplot2 (8) for general plotting, enhanced with ggpubr (Kassambara, 2020) for publication-ready figures. Color schemes were applied using viridis and viridisLite (Garnier, 2018) for perceptually

uniform color maps, RColorBrewer (Neuwirth, 2014) for qualitative and divergent palettes, wesanderson for custom palettes inspired by Wes Anderson films, and paletteer (Hvitfeldt & Whalen, 2020) for access to various color palettes. The reshape2 package (Wickham, 2007) was used to reshape and transform the data. Time series data were handled with lubridate (Grolemund & Wickham, 2011) to manage date-time data. Conflicts between packages were managed with conflict (Wickham, 2019) to ensure smooth operation of the packages. The ampvis2 package (9) was employed for amplicon data analysis, allowing easy visualization and comparison of microbiome data. All plots and figures were generated using these tools to ensure high quality and reproducible visualizations.

Alginate and Agarose artificial particles

Agarose and alginate artificial particles were prepared using protocols adapted from Enke *et al.*(2019) and Dahal *et al.* (2023). The agarose artificial particles preparation process was initiated by dissolving 1.5 g of agarose in 100 mL of Milli-Q water. The solution was then autoclaved and heated in a microwave until the agarose was completely melted. The melted agarose, totaling 20 mL, was then emulsified in 50 mL of paraffin oil preheated to 100°C by stirring the mixture in a beaker at 250 rpm. During this step, the agarose and oil were thoroughly mixed until foaming was observed. After mixing, the magnetic stirrer was removed and the beaker was closed with parafilm and immediately placed in an ice bath where the contents were gently stirred at 100 rpm on a stirring plate for 5 min to cool. The beaker was then refrigerated at 4°C for 30 min to facilitate phase separation between the oil and water. Most of the oil was then removed with a serological pipette. The remaining mixture was transferred to a 50 mL Falcon tube filled without a headspace with 0.2 µm filtered seawater, gently vortexed and centrifuged at 3000 rpm for 10 min, and the oil was discarded. This step was repeated three times until there was no more oil in the tube. The artificial particles were then washed with 50% ethanol

dissolved in filtered seawater to remove any residual oil and stored in the same solution at 4°C in a 15 mL Falcon tube until further use.

A calcium-induced polymerization process was used for the alginate artificial particles. First, a 100 mM Ca^{2+} solution was prepared by dissolving 4.4 g of anhydrous $CaCl_2$ powder in 400 mL of autoclaved Milli-Q water. Simultaneously, a 2% (w/v) alginate solution was prepared by stirring alginate powder (Merck, Darmstadt, Germany) in Milli-Q water at 300 rpm for 2 h at 100°C to ensure complete dissolution and then autoclaved. The alginate solution was then gently dispensed into the Ca^{2+} solution using a 100 µL cone-tipped pipette that allowed drops to form. Artificial particles were formed by applying constant and gentle pressure to the pipette. The artificial particles were then rinsed and stored in a 50% solution of ethanol and filtered seawater. Initial size control was performed by filtering through an 800 µm plankton mesh to remove undersized polysaccharide aggregates. Particle size distribution was initially assessed in the laboratory using a Leica M205A stereomicroscope to ensure consistent batch production. Prior to each incubation, individual particles were measured using a Dino-Lite digital microscope (200X magnification, 640 x 480 pixels; Hsinchu, Taiwan) on board, to verify the target diameter of 1 mm (Fig S6). Only particles that met these size specifications were selected for experimental use. The artificial particles were approximately 1 mm in diameter (average ± 0.15 mm)

Adaptation of the In Situ Chemotaxis Assay for particle incubations

The modified In Situ Chemotaxis Assay (ISCA) consisted of a polymethylmethacrylate 4 l container sealed with seawater-resistant silicone gaskets and a lid to maintain environmental stability during ship operations. The device featured multiple bars, each containing five wells 3D-printed with clear resin (Phrozen Aqua Resin). Each well was sealed with a lid containing a 600 µm diameter hole to allow chemical diffusion while preventing cross-contamination between samples. Single artificial particles (1 mm diameter) composed of either agarose or alginate were

placed in individual wells, with sterile glass beads of the same size serving as inorganic controls. Non-inoculated wells were included as baselines to account for the free-living microbial community fraction. This setup enabled the normalization of colonization patterns across different substrates while maintaining controlled experimental conditions throughout the cruise. Device specifications and 3D printing files are available at https://github.com/OceanBridges/In-Situ-Chemotaxis-Assay-adapted-version.

Supplementary Results

Our analysis revealed distinct colonization dynamics across particle types for both diazotrophic and total bacterial communities, highlighting the interplay between passive and active bacterial colonization mechanisms. For diazotrophs, broad colonization patterns were particle-dependent (Fig S3). While inorganic and agarose particles maintained stable colonization, alginate particles and free-living bacteria exhibited dynamic patterns. Statistical analysis confirmed this heterogeneity (Kruskal–Wallis rank sum test: $\chi^2 = 29.805$, $P < 0.01$; Fligner–Killeen test: $\chi^2 = 10.226$, $P = 0.017$) Despite the lack of significant differences in Wilcoxon rank-sum tests between organic and inorganic particles at individual timepoints, distinct colonization patterns emerged. Inorganic particles showed remarkable stability (mean fold changes: 11.8→12.2→11.9; variance: 0.305→0.463→0.764; $P = 0.79$), suggesting passive colonization. This was reinforced by consistently low residual standard deviations (0.484 ± 0.582). Agarose particles displayed similar stability in means (11.9→12.1→11.9) but with decreasing variance (0.588→0.510→0.206; $P = 0.5$). Their intermediate residual variation (1.045 ± 0.349) and R^2 values (0.176-0.507) suggest a mix of passive and active colonization, possibly influenced by carbon diffusion. Alginate particles exhibited dynamic colonization (mean fold changes: 12.1→3.73→12.3) with heterogeneous variances (0.247→2.50→0.610; $P < 0.01$). Their high residual variation (3.491 ± 5.847) indicates active bacterial responses. Free-living bacteria showed the most variability (mean fold changes: -1.89→2.75→3.44; variance: 105→1.38→2.20;

$P < 0.01$), likely due to their low abundance, resulting in the highest residual variation (7.114 ± 6.524). The total bacterial community showed different patterns (Fig. S4), did not exhibit significant heterogeneity across particle types (Fligner–Killeen: $\chi^2 = 1.9498$, $P = 0.58$). Despite the absence of significant differences in t-tests between organic and inorganic particles at any timepoint, distinct colonization dynamics were observed. Agarose particles displayed higher initial variability that decreased over time (mean fold changes: -1.59→0.350→0.539), while alginate showed moderate colonization patterns (means: -0.120→0.115→-0.517). Free-living bacteria maintained relatively stable means (-0.211→-0.211→-0.229). This pattern of increasing variability from inorganic to organic particles reveals a gradient from passive to active colonization mechanisms, with diazotrophs showing more pronounced responses than the total bacterial community, providing insights into bacterial-particle interactions in marine systems. We attempted to analyze the 16S rRNA gene data at genus level, which was not possible for *nifH* data due to limited amplification success and sequencing depth. Our multivariate analysis (PERMANOVA) showed no significant differences in genus-level community structure between substrate types ($P = 0.874$), and alpha diversity remained similar across substrates ($P = 0.591$). Although few individual genera (6 out of 179 identified) showed substrate preferences, these limited findings support our decision to conduct analyses at class level, enabling consistent taxonomic resolution between diazotrophic and non-diazotrophic communities for direct comparative analyses.

Supplementary references

1. Pontrelli S, Szabo R, Pollak S, Schwartzman J, Ledezma-Tejeida D, Cordero OX, et al. Metabolic cross-feeding structures the assembly of polysaccharide degrading communities. *Sci Adv.* 2022 Feb 25;8(8):eabk3076.

2. Callahan BJ, McMurdie PJ, Rosen MJ, Han AW, Johnson AJA, Holmes SP. DADA2: High-resolution sample inference from Illumina amplicon data. *Nat Methods.* 2016 Jul;13(7):581–3.

3. Eddelbuettel D, François R. Rcpp: Seamless R and C++ integration. J Stat Softw.

4. Wickham H, Averick M, Bryan J, Chang W, McGowan L, François R, et al. Welcome to the Tidyverse. *J Open Source Softw.* 2019 Nov 21;4(43):1686.

5. McMurdie PJ, Holmes S. phyloseq: An R package for reproducible interactive analysis and graphics of microbiome census data. Watson M, editor. *PLoS ONE.* 2013 Apr 22;8(4):e61217.

6. Oksanen J, Simpson GL, Blanchet FG, Kindt R, Legendre P, Minchin PR, et al. vegan: Community Ecology Package [Internet]. 2001 [cited 2024 Nov 20]. p. 2.6-8. Available from: https://CRAN.R-project.org/package=vegan

7. Barnett D, Arts I, Penders J. microViz: an R package for microbiome data visualization and statistics. *J Open Source Softw.* 2021 Jul 10;6(63):3201.

8. Wickham H. Getting started with ggplot2. In: ggplot2 [Internet]. *Cham: Springer International* Publishing; 2016 [cited 2024 Nov 20]. p. 11–31. (Use R!). Available from: http://link.springer.com/10.1007/978-3-319-24277-4_2

9. Andersen KS, Kirkegaard RH, Karst SM, Albertsen M. ampvis2: an R package to analyse and visualise 16S rRNA amplicon data [Internet]. 2018 [cited 2024 Nov 20]. Available from: http://biorxiv.org/lookup/doi/10.1101/299537

Supplementary tables captions

Table S1: CSV file containing the comparative table showing the average relative abundance of the four most abundant diazotroph classes in artificial agarose and alginate particles across four Barents Sea stations (N01, N12, N08, N16). The diazotroph classes included in this study are *Alphaproteobacteria*, “*Betaproteobacteria”*, *Gammaproteobacteria*, and *Cyanophyceae*. The relative abundance of each class is presented as a percentage for both agarose and alginate artificial particles, with corresponding adjusted *P* values indicating the statistical significance of differences between artificial particles and size effect. Based on the statistical results, preferences for both artificial particles are noted.

Table S2: CSV file containing the comparative table showing the average relative abundance of the four most abundant bulk microbial classes in artificial agarose and alginate particles across four Barents Sea stations (N01, N12, N08, N16). The bulk classes included in this study are *Alphaproteobacteria*, “*Betaproteobacteria*”, *Gammaproteobacteria*, and *Cyanophyceae*. The relative abundance of each class is presented as a percentage for both agarose and alginate artificial particles, with corresponding adjusted *P* values indicating the statistical significance of differences between artificial particles and the size effect. Based on the statistical results, preferences for both artificial particles are noted.

Table S3: CSV file containing the sequencing data and environmental parameters across different sample types, stations, and incubation times. The dataset includes measurements of nutrients (NN, PO4, NO2), iron (Fe), chlorophyll-a (Chl *a*), sea surface temperature (SST), photosynthetically active radiation (PAR), total incoming radiation (TIR), transmittance (Trans), and sea surface salinity (SSS). All samples were sequenced for *nifH* gene analysis.

Table S4: CSV file containing the sequencing data and environmental parameters across different sample types, stations, and incubation times. The dataset includes measurements of nutrients (NN, PO4, NO2), iron (Fe), chlorophyll-a (Chl *a*), sea surface temperature (SST), photosynthetically active radiation (PAR), total incoming radiation (TIR), transmittance (Trans), and sea surface salinity (SSS). All samples were sequenced for 16S rRNA gene analysis.

Table S5: CSV file containing the environmental DNA sequence data obtained by sequencing the 16S rRNA gene, used to characterize the composition of the microbial communities in the samples. The main columns of this table are described below: ASV_ID: Unique identifier for each Amplicon Sequence Variant (ASV). Each ASV represents a specific nucleotide sequence detected in the samples, used to identify the different operational taxonomic units (OTUs) at high resolution. Taxonomy: Taxonomic classification of each ASV. This column can include classification levels such as Domain, Phylum, Class, Order, Family, Genus, and possibly Species, if available. XSample.16S : Columns representing the individual samples collected.

Table S6: CSV file containing the DNA sequence data obtained by sequencing the *nifH* gene, used to characterize the composition of the microbial communities in the samples. The main columns of this table are described below: ASV_ID: Unique identifier for each Amplicon Sequence Variant (ASV). Each ASV represents a specific nucleotide sequence detected in the samples, used to identify the different operational taxonomic units (OTUs) at high resolution. Taxonomy: Taxonomic classification of each ASV. This column can include classification levels such as Domain, Phylum, Class, Order, Family, Genus, and possibly Species, if available. XSample.nifH : Columns representing the individual samples collected.

Table S7: CSV file containing the number of samples per station, incubation time and substrate type for analysis of the *nifH* gene. Columns represent: Station (sampling site), Incubation (incubation time in hours), Type (substrate category or condition - Inorganic, Agarose, Alginate, or Free Living), and n (number of biological replicates). Samples were collected at four stations (N01, N08, N12 and N16) with three incubation times (2h, 36h, and 72h) for four different types of conditions.

Table S8: CSV file containing the number of samples per station, incubation time and substrate type for 16S rRNA gene analysis. Columns represent: Station (sampling site), Incubation (incubation time in hours), Type (substrate category or condition - Inorganic, Agarose, Alginate, or Free Living), and n (number of biological replicates). Samples were collected at four stations (N01, N08, N12 and N16) with three incubation times (2h, 36h, and 72h) for four different types of conditions.

Supplementary figures captions

Figure S1: Chlorophyll *a* concentration (μg L^{-1}) across the Barents Sea on June 1, 2023, based on satellite data from the TOPAZ5-ECOSMO Arctic operational system. The map shows a major phytoplankton bloom with chlorophyll *a* concentrations reaching up to 10 μg L^{-1} in the sampling region. Red dots represent sampling stations (N01, N08, N12, N16) across the study area. Data visualization was created in R. Source: CMEMS model ECOSMO.

Figure S2: Temperature-salinity (T-S) diagram showing water mass properties at sampling stations. Colors indicate beam transmission (%), with warmer colors (yellow) representing higher transmission (90-100%) and cooler colors (blue) showing lower transmission (0-40%). Sampling stations (N01, N08, N12, N16) are marked by black dots, with N01 in Atlantic Water (AW), and stations N08, N12, and N16 in warm Polar Water (wPW). Grey open circles represent CTD bottle data from all sampled depths. Black lines delineate water mass boundaries following Sundfjord et al. (2020): Atlantic Water (AW), warm Polar Water (wPW), modified Atlantic Water (mAW), Intermediate Water (IW), and Cold Bottom Surface Water Derived (CBSD). The dashed line indicates the freezing point at atmospheric pressure.

Figure S3: Diazotrophs average fold change trends by particle type, all stations, and incubation times combined from the *nifH* data. The colored lines indicate the average trend for each bacterial class (*Alphaproteobacteria*, "*Betaproteobacteria*", *Cyanophyceae*, and *Gammaproteobacteria*). The grey shading around the dotted lines represents the 95% confidence interval for the mean trend. Fold changes are presented on a base-2 logarithmic scale (log2) to enable a fair comparison of increases and decreases. The symbols represent individual measurements for each sampled station. Detailed information on individual samples is provided in Tables S7 and S8.

Figure S4: Bulk microorganisms average fold change trends by particle type, all stations, and incubation times combined from the 16S rRNA gene data. The dotted lines indicate the average trend for each particle type. The colored lines indicate the average trend for each bacterial class (*Alphaproteobacteria*, “*Betaproteobacteria*”, *Cyanophyceae*, and *Gammaproteobacteria*). The gray shading around the dotted lines represents the 95% confidence interval for the mean trend. Fold changes are presented on a base-2 logarithmic scale (log2) to enable a fair comparison of increases and decreases.The symbols represent individual measurements for each sampled station. Detailed information on individual samples is provided in Tables S7 and S8.

Figure S5: Schema of the In Situ Chemotaxis Assay (ISCA) device, illustrating its key components utilized for microbial incubations and sampling. The ISCA is designed for in situ deployment, which allows for the study of microbial chemotaxis in response to different chemical gradients within an aquatic environment.

Figure S6**:** Comparative microscopic observation of artificial particles of different compositions. (a) Alginate beads, (b) agarose beads, and (c) glass beads (control). Images acquired with a Dino-Lite digital microscope (200X magnification, 640 x 480 pixels; Hsinchu, Taiwan). The four points of light visible in each image correspond to the microscope illumination. Scale bar: 1 mm.

Figure S7: Heatmaps showing relative abundance (% Read Abundance) of microbial communities across four sampling stations (N01, N08, N12, N16) in the Barents Sea. a) *nifH* gene abundance representing nitrogen-fixing potential and b) 16S rRNA gene abundance representing overall bacterial community composition in free-living samples. Samples were collected at different time points (2h, 36h, 72h) to characterize the microbial seed bank at each station. Taxonomic classes in the 16S rRNA gene dataset are arranged in the same order as

the *nifH* dataset to improve visibility and facilitate comparison between the two gene markers. The number of replicates is indicated at the bottom of the heatmap and detailed information on individual samples is provided in Tables S7 and S8.

Figure S8: Normalized relative abundance of diazotrophs families over time in agarose (a,c,e,g) and alginate (b, d, f, h) artificial particles at four Barents Sea stations (N01, N08, N12, N16). The diazotroph families include representatives of the *Gammaproteobacteria*, *Cyanophyceae*, “*Betaproteobacteria*”, and *Alphaproteobacteria*. The time points included in the study were 0, 2, 36, and 72 h. Bootstrap confidence intervals (95%) are displayed as semi-transparent shaded areas around each curve. The number of samples included in each data point are provided in Table S7.
